# Evidence of the use of the Perme Intensive Care Unit Mobility Score in hospitalized adults: a scoping review

**DOI:** 10.1590/1518-8345.7491.4542

**Published:** 2025-05-02

**Authors:** Maria Helena Lenard, Clovis Cechinel, Tissiane Bona Zomer, João Alberto Martins Rodrigues, Maria Angélica Binotto, Rossana Spoladore

**Affiliations:** 1 Universidade Federal do Paraná, Curitiba, PR, Brazil; 2 Hospital Municipal Zilda Arns, Curitiba, PR, Brazil; 3 Scholarship holder at the Ministério da Saúde, Secretaria de Gestão do Trabalho e da Educação na Saúde, Programa de Residência em Saúde do Idoso, Brazil; 4 Universidade Estadual do Centro-Oeste, Departamento de Educação Física, Irati, PR, Brazil

**Keywords:** Mobility Limitation, Early Ambulation, Physical Therapy Modalities, Inpatients, Rehabilitation Nursing, Review

## Abstract

to map the scientific literature regarding the use of the Perme Intensive Care Unit Mobility Score in hospitalized adults.

scoping review, structured according to the methodological guidelines of the Joanna Briggs Institute - Evidence Synthesis Groups, with searches in seven databases and gray literature. The studies were selected by two reviewers, using an instrument for data extraction.

the analysis of the 29 selected studies showed a predominance of longitudinal studies (34.48%), conducted in Brazil (48.27%) in Intensive Care Units (29%), and published between 2020 and 2021 (48.24%). The studies demonstrated the use of the Perme Score for description and reliability of the instrument, translation and cultural adaptation, association between functional mobility, clinical characteristics and outcomes, mobility assessment after interventions, mobility assessment and potential barriers to mobilization, and use of the score for validation of other instruments and various clinical profiles.

the Perme Score is an instrument capable of measuring physical mobility, including possible barriers to mobility, with potential for use in scenarios outside the Intensive Care Unit, in intervention studies for early mobilization and prediction of hospitalization outcomes.

## Highlights

**(1)** The Perme Score demonstrates great potential for use in various settings. **(2)** Studies position nurses within the context of functional mobility assessment. **(3)** The Perme Score has been employed in intervention protocols for early mobility.

## Introduction

The hospital institution is considered a space that provides symptom relief, health recovery, and access to diagnosis. However, depending on the care and treatments provided, as well as other factors, hospitalization can become a disruptive factor, leading to greater clinical and functional deterioration, increased length of stay, decompensation of multimorbidities, and higher risk of mortality^(1)^.

The consequences of mobility decline during hospitalization can extend for up to five years after discharge, resulting from prolonged hospitalizations associated with age, disease severity, and type of admission (acute/elective). These were the conclusions of a cohort study conducted with 10,430 individuals, in the Intensive Care Unit (ICU) group (n= 5,215) and in the general ward group (n= 5,215), with a median age of 60 years (range 44–72) in Edinburgh (United Kingdom). The ICU group showed an association with higher mortality (RR 1.33; 95% CI, 1.22–1.46, p=0.001), higher costs ($25,608 vs. $16,913/patient^1^), and longer hospital stay (51%)^(2)^.

It is estimated that hospitalization is associated with a 30% physical deconditioning^(3)^. Therefore, functional decline is not only related to the clinical condition that led to hospitalization, and recovery is not automatic after the resolution of the issue that caused it^(4)^. It is important to note that functional mobility is a predictor of health. Thus, assessing locomotor functions in the hospital context and understanding the barriers to early mobilization become essential.

Early mobilization should be understood as part of the rehabilitation process of hospitalized patients, especially in the ICU, minimizing muscle weakness and worsening of physical function^(5)^. There are several scales that assess functional aspects in the ICU, such as the Physical Function in Intensive care Test scored, Chelsea Critical Care Physical Assessment tool, Perme Intensive Care Unit Mobility Score, Surgical intensive care unit Optimal Mobilization Score, ICU Mobility Scale and Functional Status Score for the ICU^(6)^. None of them have been considered the “gold standard” for quantifying functional mobility, while also being quick and objective to apply. However, the Perme Intensive Care Unit Mobility Score takes into account extrinsic conditions affecting the patient’s mobility in bed, such as the presence of access lines, tubes and chest drains, which can be interpreted as barriers to mobility. The presence of these devices is not scored or considered in most scales.

The Perme Intensive Care Unit Mobility Score, created by Christiane Strambi Perme and here referred to as the Perme Score, was translated and adapted into Portuguese. It consists of seven categories that assess mental state, potential barriers to mobility, functional strength, bed mobility, transfers, assistive devices for ambulation, and resistance measures. The score ranges from 0 to 32 points; a higher score indicates greater mobility and less need for assistance, while a lower score indicates lower mobility and greater need for assistance^(7)^.

Despite being a relatively new instrument, already translated into different languages, and introducing the innovation of assessing potential barriers to mobility in the ICU, it is essential to represent publications on the score in the literature, considering different clinical conditions and settings. After a preliminary search in public databases of review protocol registrations and online databases, and in the absence of systematic reviews or protocol records on this scale, the development of this review was deemed essential. The aim was to map the scientific literature regarding the use of the Perme Intensive Care Unit Mobility Score in hospitalized adults.

## Method

### Type of study

This is a scoping review, a systematic method that identifies and synthesizes knowledge through existing or emerging literature, maps the extent and scope of the topic, the nature of the literature, and identifies potential gaps^(8)^. The review was developed according to the recommendations of the JBI – Evidence Synthesis Groups, a method recommended for scoping reviews^(9)^, in addition to the guidelines set by the Preferred Reporting Items for Systematic Reviews and Meta-Analyses Extension for Scoping Reviews (PRISMA-ScR), and was registered in the International Platform of Registered Systematic Review and Meta-analysis Protocols (INPLASY), under number 2023100031 (https://inplasy.com/inplasy-2023-10-0031).

Thus, the scoping review was operationalized in five stages: 1- establishment of the research question; 2- identification of relevant studies; 3- selection and inclusion of studies; 4- data organization; and 5- collection, synthesis and reporting of results^(10-11)^. The question and the main search elements for this review were developed using the PCC strategy (P – Population or Patients; C – Concept; C – Context)^(12)^, with P (adult individuals), C (Perme Intensive Care Unit Mobility Score), and C (hospital). In Context, hospital environment refers to any care sector within the hospital unit.

Therefore, the following question was formulated: what is the available evidence in the literature regarding the use of the Perme Intensive Care Unit Mobility Score in hospitalized adults?

### Selection criteria

Regarding the eligibility of the studies, the inclusion criteria were: (1) to be primary and secondary empirical research, both quantitative and qualitative, of any design or methodology; (2) to include the variables of interest “Perme Score” and “hospitalization”; (3) to be published in English, Spanish, Portuguese or French; (4) to have been published since 2014, the year the scale was published. The exclusion criteria for the studies were: letters to the editor, abstracts in conference proceedings, dissertations, theses, monographs, and case reports that did not present the variable of interest of the research.

### Data search and collection

Initially, the search strategy was developed based on the identification of the Medical Subject Headings (MeSH) descriptors: mobility limitation, early ambulation, physical therapy modalities, and inpatients, associated with the free term “Perme”. These descriptors were then translated into the specific terms for each database searched, such as the Health Sciences Descriptors (DeCS) and the Embase Subject Headings (Emtree).

However, the databases and portals tended to zero when the descriptors were combined using the Boolean operator “AND”. Consequently, assistance from a professional librarian was sought to minimize the possibility of errors. Thus, the search strategy was redefined to include only the free terms: “Perme” OR “Perme scale” OR “Perme score”.

In October 2023, searches were conducted in the following databases: Virtual Health Library*,* EMBASE, PEDro, PubMed, SciELO, Scopus and Web of Science. The titles and abstracts of the articles were sent to two reviewers, who independently assessed their eligibility. For gray literature, the search was conducted in the Catalog of Theses and Dissertations of the *Coordenação de Aperfeiçoamento de Pessoal de Nível Superior* (CAPES). Among the gray literature studies that met the inclusion and exclusion criteria, all already had articles published in journals, which were included in the review.

The results of the textual searches were exported and transferred to the free reference manager Mendeley^U+00AE^, a tool that allows access by multiple researchers and organizes references into separate folders. Duplicates were removed, keeping only one of the titles. Subsequently, the titles and abstracts were reviewed, and articles that did not meet the inclusion criteria or contained any exclusion criteria were eliminated. Finally, the selected texts were made available to the reviewers for full reading, completing the inclusion process.

The researchers, responsible for the review, are professionals with expertise in gerontology and members of a Multidisciplinary Research Group on the Elderly.

It is worth noting that the Kappa concordance coefficient was used to describe the degree of agreement between the reviewers, which is based on the number of concordant responses, i.e., the frequency with which the results coincide between reviewers^(13)^. In this study, the overall agreement among reviewers was 96.81%, corresponding to a Kappa coefficient higher than 0.90. Furthermore, a third reviewer assessed the discrepancies in study selection to make the final decision on inclusion or exclusion, aiming to minimize the risk of bias.

The number of articles found in each database and the sum of all databases were recorded in the PRISMA flow diagram^(14)^, as well as the selection process and reasons for exclusion.

### Data extraction and analysis

The extraction and descriptive analysis of the data were conducted using a protocol developed by the authors themselves, which encompasses the previously defined eligibility criteria, as widely recommended by the JBI. In this context, the following information was included: author’s name and year of publication, journal, country of origin, objectives, results, study design and sample size.

Due to the chosen method, there was no need for a formal assessment of the methodological quality of the included studies. However, adherence to the evaluation items of the PRISMA Extension for Scoping Reviews (PRISMA-ScR) checklist aimed to ensure the methodological rigor of the content^(15)^. Finally, a thematic analysis of the content was conducted to identify the converging points in the literature, outline the strengths of the topic, and highlight existing gaps.

### Ethical aspects

Since the studies used are in the public domain, there was no need for submission to the Research Ethics Committee, according to Resolution CNS n° 510, of 2016.

## Results

**Figure 1 -:**
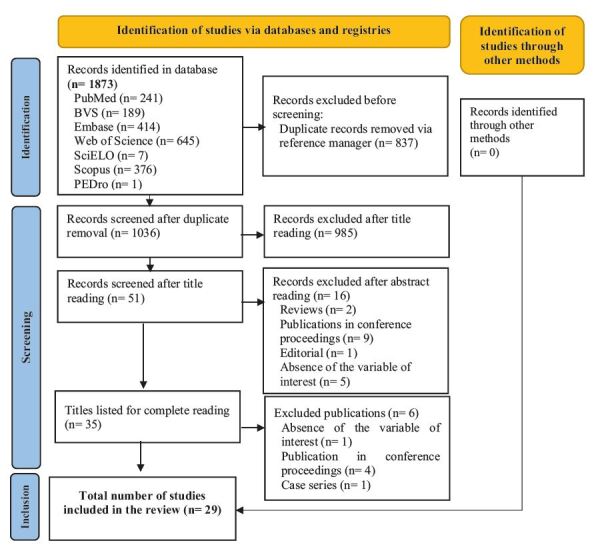
PRISMA-ScR flow diagram of study selection. Curitiba, PR, Brazil, 2024

The search in the portals and databases resulted in 1,873 studies. Of these, 837 were excluded as duplicates, using the free reference manager Mendeley^U+00AE^. After removing the duplicates, the 1,036 articles selected for title and abstract reading were organized in an Excel^U+00AE^spreadsheet for further analysis. Subsequently, 985 articles were excluded after reading the titles, and 16 after reading the abstracts, resulting in 35 studies for full-text rerganized in an Excelview. Of these, six were excluded, totaling 29 studies for review. To minimize the possible risk of bias in the selection of studies, the reviewers organized the references in the free reference manager Mendeley^U+00AE^. The refinement was conducted by two independent evaluators aiming for 100% agreement. A third reviewer assessed any potential discrepancies in the selection of studies to make the final decision on inclusion or exclusion. These procedures are represented in Figure 1, in the Preferred Reporting Items for Systematic Reviews and Meta-Analysis (PRISMA) flow diagram, which illustrates the article selection process for this review^(14)^.

**Figure 2 -:**
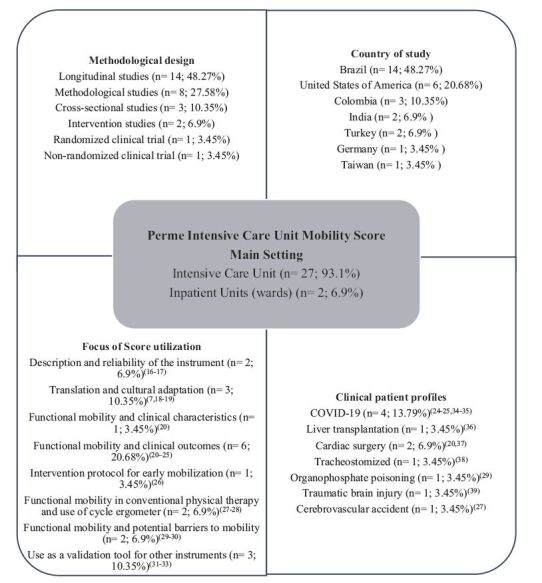
Schematic representation of the findings of studies involving the Perme Intensive Care Unit Mobility Score. Curitiba, PR, Brazil, 2024

In Figure 2, the methodological design, the study focus, the study locations, and the clinical profiles, which are the main findings, are observed.

In Figure 3, the articles are categorized by author/year of publication, journal, country of origin, objectives, results, study design, and sample size.

Among the studies analyzed, most publications were from 2021 (n= 8; 27.58%), followed by 2020 (n= 7; 24.14%), 2022 and 2023 (n= 3; 10.34% each year), 2014, 2018 and 2019 (n= 2; 6.9% each year), and finally, 2016 and 2017 (n= 1; 3.45% each year).

Regarding the journals, 25 different ones were identified, with a predominance of *Colombia Médica*, NeuroRehabilitation, PloS One and *Revista Brasileira de Terapia Intensiva* (n= 2; 6.89% each). The countries that stood out as host of the studies were: Brazil (n= 14; 48.27%), United States of America (n= 6; 20.68%), Colombia (n= 3; 10.35%), India and Turkey (n= 2; 6.9% each), in addition to Germany and Taiwan (n= 1; 3.45% each). All articles (n= 29) were published in English.

Regarding the methodological design, longitudinal studies predominated (n= 10; 34.48%). The others were methodological studies (n= 8; 27.58%), retrospective cohort studies (n= 4; 13.79%), cross-sectional studies (n= 3; 10.35%), interventional studies (n= 2; 6.9%), randomized clinical trial and non-randomized clinical trial (n= 1; 3.45% each). A total of 3,399 participants were observed in the studies, with sizes ranging from 18 to 949 participants.

**Figure 3 –:** Characterization of the studies included in the scoping review (n = 29). Curitiba, PR, Brazil, 2024

**Author/ Year**	**Journal**	**Country of origin**	**Objectives**	**Results**	**Study design**	**Sample size**
Perme, et al., 2014 ^( 16 )^	Methodist DeBakey Cardiovascular Journal	USA*	To describe a tool that assesses ICU ^†^ mobility and reliability, and to address clinical application.	- Perme Score ^‡^ consisting of 15 items grouped into the following categories: mental status, potential mobility barriers, functional strength, bed mobility, transfers, gait, and endurance;	Methodological study	35
				- Kappa agreement between evaluators: 94.29% (68.57%-100%). The Kappa values were: item 2 (mental status: patient can follow 2 out of 3 commands), no agreement (κ= 0); item 1 (alertness during initial contact), fair agreement (κ= 0.21-0.40); items 5, 10, 12 and 13, moderate agreement (κ= 0.41-0.60); items 4, 7, 9 and 11, substantial agreement (κ= 0.61-0.80); and items 3, 6, 8, 14 and 15, high agreement (κ= 0.81-1.00).		
Nawa, et al., 2014 ^( 17 )^	Journal of Critical Care	USA*	To determine the inter-rater reliability of the Perme Score ^‡^ in patients admitted to the Cardiovascular ICU ^†^ .	The 15 items of the score were analyzed individually. The inter-rater reliability was as follows: 1.0 for items 2, 3, 5, 6, 7, 8, 13 and 15; 0.82 for item 1; 0.80 for item 4; 0.60 for item 9; 0.72 for items 10 and 11; 0.78 for item 12; and 0.90 for item 14. The intraclass correlation coefficient was 0.98 (95% CI 0.97-0.99).	Methodological study	20
Kawaguchi, et al., 2016 ^( 7 )^	*Jornal Brasileiro de Pneumologia*	Brazil	To translate, adapt and validate the Perme Score ^‡^ and IMS ^§^ for Brazilian Portuguese.	The instruments showed excellent interobserver agreement (κ> 0.90) and reliability (α> 0.90) in all domains. There was a strong positive correlation between the two instruments (r= 0.941; *p* < 0.001).	Methodological study	103
Yang, et al., 2017 ^( 21 )^	American Journal of Respiratory and Critical Care Medicine	USA*	To identify the Perme Score ^‡^ of patients at ICU ^†^ discharge and the final discharge destination.	The median Perme Score ^‡^ for patients discharged home was 29; home care, 12; rehabilitation hospital, 26; skilled nursing facility, 13; and death, 7. Higher Perme Score ^‡^ values were associated with home discharge ( *p* < 0.05).	Prospective longitudinal study	157
Nydahl, et al., 2018 ^( 18 )^	European Journal of Physiotherapy	Germany	To translate the Perme Score ^‡^ into German and investigate the inter-rater reliability between physiotherapists and nurses.	The inter-rater reliability (nurses and physiotherapists) was nearly perfect: 0.96 (95%CI: 0.93-0.97). Two items were assessed with a reliability index lower than 0.8: ability to follow commands (0.73, 95%CI: 0.55-0.89) and pain (0.46, 95%CI: 0.09-0.68). The scale completion time was approximately 30 seconds. This is the first study comparing its application across different professionals.	Methodological study	58
Wilches Luna, et al., 2018 ^( 19 )^	*Colombia M* é *dica*	Colombia	To perform the translation, cultural adaptation and inter-rater reliability of the Spanish versions of the Perme Score ^‡^ and IMS ^§^ in ICU ^†^ patients.	The inter-rater reliability of the IMS ^§^ ranged from 0.97 to 1.0, and for the Perme Score ^‡^ it was between 0.99 and 1.0 at both measurement points, up to 24 hours after admission and ICU ^†^ discharge.	Methodological study	150
Moecke & Biscaro, 2019 ^( 22 )^	*Fisioterapia Brasil*	Brazil	To analyze the functional status of patients admitted to the ICU ^†^ and associate it with functional and clinical outcomes, and muscle strength.	- Perme Score ^‡^ : 8.18±3.99 upon awakening from sedation and 13.68±6 upon discharge from the ICU ^†^ ;	Prospective longitudinal study	40
				- Functional status and frailty are associated with clinical outcomes (discharge/transfer and death) ( *p* = 0.022 and *p* = 0.019);		
				- MRC ^||^ was associated with Perme Score ^‡^ upon awakening ( *p* < 0.001) and at ICU ^†^ discharge ( *p* = 0.002);		
				- Higher Perme Score ^‡^ values are associated with MRC ^||^ > 48.		
Pereira, et al., 2019 ^( 36 )^	*Revista Brasileira de Terapia Intensiva*	Brazil	To evaluate the predictive power of the Perme Score ^‡^ for complications in the postoperative period of liver transplantation.	- Perme Score ^‡^ at ICU ^†^ admission (5.5) and discharge (23.6), in the ward 28.2±5, and at hospital discharge 31.7±0.7;	Prospective observational study	30
				- When comparing the initial assessment in the ward and at hospital discharge, there was an improvement in functional status: Perme Score ^‡^ 28.2 ± 5 to 31.7 ± 0.7, *p* < 0.001;		
				- The duration of MV ^¶^ was associated with the Perme Score ^‡^ at ICU ^†^ discharge (r = -0.374; *p* = 0.042). The longer the MV ^¶^ , the lower the Perme Score ^‡^ at ICU ^†^ discharge;		
				- The number of physiotherapy treatments was inversely associated with the Perme Score ^‡^ at the IU ^**^ (r = -0.578; *p* = 0.001).		
Ozcan, et al., 2020 ^( 31 )^	Disability and Rehabilitation	Turkey	To translate and cross-culturally adapt the FSS-ICU ^††^ instrument into Turkish and evaluate its psychometric properties.	- Internal consistency was high (Cronbach’s α=0.949). Inter- and intra-rater reliability was excellent (α= 0.955-0.996);	Methodological study	50
				- FSS-ICU ^††^ score showed moderate to high correlations with other functional measures, such as: Perme Score ^‡^ (Spearman’s r= 0.92), Katz activities of daily living (r= 0.80) and handgrip strength (r= 0.76–0.77).		
Cavalli, et al., 2020 ^( 40 )^	Middle East Journal of Rehabilitation and Health Studies	Brazil	To analyze the discharge or mortality outcomes of ICU ^†^ patients considering age, sex, severity, reason for admission, comorbidities, and length of stay in the ICU ^†^ and hospital.	- The mean age was 54.91±19.7 years, with 62.5% male participants, a predominance of neurological conditions (34%), on MV ^¶^ (63,9%), a mean ICU ^†^ stay of 5.76 days with an initial Perme Score ^‡^ of 4.48 ± 7.17. The initial Perme Score ^‡^ was lower in cases of death (0.57±1.98) compared to hospital discharge (7.27±8.16), *p* < 0.0001. Furthermore, the use of vasoactive and sedative drugs was higher in cases of death, *p* ≤ 0.0001;	Cross-sectional study	288
				- There was a significant difference in the initial Perme Score ^‡^ based on the type of admission: medical patients 4.15±7.30 and surgical patients 5.44±6.75, *p* <0.01.		
Ceron, et al., 2020 ^( 38 )^	Respiratory Care	Brazil	To evaluate mobility performance changes with the use of speaking valves in tracheostomized individuals.	- Perme Score ^‡^ increased from 11.3 (IQR ^‡‡^ 10.1-12.0) to 18.2 (IQR ^‡‡^ 16.2-20.1) immediately after the initiation of the speaking valve, *p* < 0.01.	Prospective cohort study	18
				- When evaluating the Perme Score ^‡^ categories, changes were observed in the scores of the “transfer category” (“sitting to standing”, “static standing balance once the standing position is established” and “transferring from bed to chair or chair to bed”);		
				- The use of speaking valves in tracheostomized patients improved mobility.		
Gatty, et al., 2020 ^( 26 )^	Physiotherapy Theory and Practice	India	To study the effectiveness of an early mobilization protocol on the mobility status of ICU ^†^ patients	- Perme Score ^‡^ on the first ICU ^†^ day, median (IQR ^‡‡^ ): intervention group 6 (2.2-8) and control group 6 (3-7); on the first day of rehabilitation, (IQR ^‡‡^ ): intervention group 7 (5-9) and control group 5 (3-8); and on the last day of rehabilitation, (IQR ^‡‡^ ): intervention group 18 (11-24.7) and control group 7 (5-11);	Non-randomized clinical trial	63
				- The difference in Perme Score ^‡^ values between the first and last day of rehabilitation was 9 (3.2-17) in the intervention group and 2 (0-5) in the control group, *p* ≤ 0.001;		
				- There was a significant increase in the median Perme Score ^‡^ from the first day of ICU ^†^ admission to the last day of rehabilitation: 12.5 (6.2–7.7) in the intervention group and 2 (0–6) in the control group, *p* < 0.001;		
				- The Perme Score ^‡^ was compared between the first day of ICU ^†^ and the first day of rehabilitation, and no difference was observed in the intervention group ( *p* = 0.069) or the control group ( *p* = 0.124).		
Lima, et al., 2020 ^( 23 )^	*Fisioterapia em Movimento*	Brazil	To determine the relationship between functional mobility and clinical outcomes of patients admitted to the ICU ^†^ .	- Clinical outcomes (death/discharge) were associated with the Perme Score ^‡^ : mental status ( *p* = 0.040), mobility barriers ( *p* = 0.016), strength ( *p* = 0.01) and bed mobility ( *p* = 0.024). The total Perme Score ^‡^ was 0 (0-10) for death and 10 (0-23) for discharge, ( *p* = 0.002);	Prospective longitudinal study	33
				- The use of MV ^¶^ was associated with low scores on the scale, 2,40 ± 4,19, while its absence was associated with higher scores, 12,85±6,61, *p* = 0.000;		
				- There was a positive correlation (R= 0.745) between Perme Score ^‡^ and sedation level, and an inverse correlation between APACHE ^§§^ and Perme Score ^‡^ (R= -0.526), as well as between APACHE ^§§^ and days of MV ^¶^ (R= -0.602).		
Perme, et al., 2020 ^( 41 )^	Journal of Acute Care Physical Therapy	USA*	To evaluate the association between the Perme Score ^‡^ instruments, the MRC-SS ^||||^ and clinical outcome of patients admitted to the ICU ^†^ .	- The average Perme Score ^†^ was 23.56 (±7.09);	Prospective longitudinal study	250
				- The Perme Score ^†^ by discharge destination was: home 26.05±5.42, long-term care facilities 18.65±8.43, specialized nursing services 17.38±7.72, rehabilitation 20.3±7.48, and others 18±5.8;		
				- The Perme Score ^‡^ of discharged patients was different from those discharged to rehabilitation ( *p* < 0.001), specialized nursing service ( *p* < 0.001), long-term acute care ( *p* < 0.001) and palliative/hospice care or deceased ( *p* < 0.001);		
				- There is a moderate correlation between the MRC-SS ^||||^ and the Perme Score ^‡^ (r= 0.66; *p* < 0.001);		
				- ICU ^†^ patients with a higher Perme Score ^‡^ or MRC-SS ^||||^ at the time of physical therapist assessment were discharged home, while those with lower scores required post-acute care;		
				- A higher Perme Score ^‡^ or MRC-SS ^||||^ indicated a greater likelihood of discharge home.		
Pinto, et al., 2020 ^( 29 )^	Critical Reviews™ in Physical and Rehabilitation Medicine	India	To Identify PBM ^¶¶^ and assess changes in mobility during ICU ^†^ stay in patients with organophosphate poisoning.	- The Perme Score ^‡^ (2.50-6.0; *p* ≤ 0.01) improved in the subcomponents bed mobility (1.83±0.35 to 7.23±3.84), transfers (0.37±0.02 to 4.78±3.84) and gait (1.83±0.35 to 5.23±1.27) from day 2 to day 10 of hospital admission;	Cross-sectional study	37
				- Endotracheal tube and continuous drug infusion were identified as PBM ^¶¶^ (66.6%) by the Perme Score ^‡^ .		
Wilches Luna, et al., 2021 ^( 42 )^	Physiotherapy Research International	Colombia	To determine MDC ^***^ and responsiveness of the Perme Score ^‡^ in adults admitted to the ICU ^†^ .	- The MDC ^***^ value for the Perme Score ^‡^ was 1.36, demonstrating that adults admitted to the ICU ^†^ who had a difference between the first and second scores greater than 1.36 points had a minimal detectable difference;	Prospective longitudinal study	142
				- MDC ^***^ occurred in 80% of patients;		
				- There was a significant difference in the duration of MV ^¶^ (0.011) and in the ICU ^†^ length of stay ( *p* < 0.04).		
Cordeiro, et al., 2021 ^( 37 )^	Journal of Clinical and Translational Research	Brazil	To evaluate the association between early ambulation and functionality in patients undergoing heart valve replacement surgery.	- The walking group had a decrease of 11±2 in Perme Score ^‡^ , while the non-walking group had a decrease of 13±2, *p* = 0.34.	Prospective cohort study	170
Da Rosa, et al., 2021 ^( 27 )^	NeuroRehabilitation	Brazil	To verify the association between cycling aerobic training and lower limb muscle strength, walking speed, balance, mobility and functionality in individuals with CVA ^†††^ .	- Intervention: cycling aerobic training;	Randomized clinical trial	20
				- In the analysis of lower limb muscle strength, there was intergroup improvement between pre- and post-intervention. Significant improvement was observed in all muscle groups, including both the paretic and non-paretic sides, only in IG ^‡‡‡^ ;		
				- In the 10mWT ^§§§^ and in the BBS ^||||||^ there was an intragroup difference in IG ^‡‡‡^ , *p* < 0.001, and an intergroup difference with a better result for IG ^‡‡‡^ *p* < 0.001. It was observed that IG ^‡‡‡^ showed improvement in balance (pre 0 ± 0, post 28.9 ± 7.45) and in gait speed (pre 0 ± 0, post 0.67 ± 0.78);		
				- Regarding the Perme Score ^‡^ , there was a difference between the results of the IG ^‡‡‡^ (pre 14.3 ±3.10 and post 27.3 ± 2.91, *p* < 0.001) and the CG ^****^ (pre 9.40±2.31 and post 12.30±3.40, *p* < 0.001).		
				- Greater mobility was observed in individuals after an acute CVA ^†††^ who underwent cycle ergometer training.		
Luna, Perme e Gastaldi, 2021 ^( 30 )^	Canadian Journal of Respiratory Therapy	Colombia	To identify early PBM ^¶¶^ in adults using the Perme Score ^‡^ and association with days on MV ^¶^ and ICU ^†^ length of stay.	- There were inverse correlations between the total number of days on MV ^¶^ and the total PBM ^¶¶^ score at ICU ^†^ admission (r = -0.773; *p* < 0.05) and discharge (r= -0.559; *p* < 0.05) as well as between ICU ^†^ length of stay and the total PBM ^¶¶^ score at admission (r = -0.420; *p* < 0.05) and discharge (r= 0.283; *p* < 0.05);	Prospective observational study	142
				- There was a correlation between the total score of the barriers item and the total Perme Score ^‡^ (r= -0.91; *p* < 0.01).		
Özsoy, et al., 2021 ^( 32 )^	Turkish Journal of Medical Sciences	Turkey	To translate and culturally adapt the IMS ^§^ into Turkish and investigate its psychometric properties.	- The inter- and intra-rater reliability of the IMS ^§^ was excellent, with a Kappa index of 0.87 (0.80-0.93) and 0.92 (0.87-0.96);	Methodological study	70
				- There are significant correlations between the IMS ^§^ scale and the FSS-ICU ^††^ , Perme Score ^‡^ , Katz, right and left HGS ^††††^ (rs ≥0.60; *p* < 0.05);		
				- ICU ^†^ environment, mean age 69.65±10.73, 60% male, main diagnosis at admission was acute coronary syndrome 54.3%, Perme Score ^‡^ 21.32±5.09.		
Souza, et al., 2021 ^( 28 )^	*Revista Pesquisa em Fisioterapia*	Brazil	To evaluate the energy and protein supply of critically ill patients undergoing conventional physiotherapy combined with an active cycle ergometer (IG ^‡‡‡^ ) or conventional physiotherapy (CG ^****^ ) and correlate with MRC ^||^ , anthropometric data and Perme Score ^‡^ .	- The level of caloric and protein adequacy in both groups was 73.9% and 69.5%, respectively. In CG ^****^ caloric adequacy was 66.6% and protein adequacy was 58.3%, while in IG ^‡‡‡^ the adequacy was 81.8% for both caloric and protein intake;	Interventional study	23
				- There was a significant reduction in calf circumference on day 9 compared to the baseline, *p* = 0,001. No difference was observed between the CG ^****^ and the IG ^‡‡‡^ , p= 0.053);		
				- There was a significant reduction in arm circumference on day 9 compared to the baseline in both groups (CG ^****^ *p* = 0.038 and IG ^‡‡‡^ *p* = 0.041);		
				- A moderate inverse correlation was found between energy deficit and the final Perme Score ^‡^ in the CG ^****^ (r= 0.59; *p* = 0.03).		
Timenetsky, et al., 2021 ^( 34 )^	PLoS One	Brazil	To describe the mobility level of COVID-19 patients admitted to the ICU ^†^ and address factors associated with the mobility level at the time of ICU ^†^ discharge.	- The Perme Score ^‡^ improved when comparing discharge 20.0 (7-28) to admission 7.0 (0-16) in the ICU ^†^ , *p* < 0.001;	Retrospective cohort study	136
				- There was an improvement in mobility during ICU ^†^ admission in 64.7% of patients, and the median Perme Score ^‡^ was 1.5 (0.6-3.4);		
				- The improved group had a shorter duration of MV ^¶^ : 10 (5-14) vs. 15 (8-24) days, *p* = 0.021; shorter hospital stay: 25 (12-37) vs. 30 (11-48) days, p< 0.001; and lower ICU ^†^ and hospital mortality;		
				- Independent predictors for mobility were younger age, lower Charlson Comorbidity Index, and not having received renal replacement therapy.		
Reis, et al., 2021 ^( 33 )^	*Revista Brasileira de Terapia Intensiva*	Brazil	To translate and cross-culturally adapt the Early Rehabilitation Index into Brazilian Portuguese and verify the psychometric properties of the tool (ERBI ^‡‡‡‡^ ) at ICU ^†^ discharge.	- The ERBI ^‡‡‡‡^ had adequate reliability, with a Cronbach’s alpha coefficient of 0.65;	Methodological study	122
				- Inter-rater reliability was excellent, with a coefficient of 0.94 (95%CI 0.92-0.96);		
				- The validity of the ERBI ^‡‡‡‡^ was observed through strong and significant correlations with the total Perme Score ^‡^ (rô= 0.72);		
				- ICU ^†^ characteristics: Perme Score ^‡^ 25.5 (15-30), mean age 56 years (46.8-66), male 51%, reason for admission (23% sepsis, 19% elective postoperative, 14% cardiovascular disorder), 68% MV ^¶^ , VM ^¶^ duration 5 (3-8) days, and hospital discharge 88%.		
Nawa, et al., 2022 ^( 20 )^	*Colombia M* é *dica*	Brazil	To evaluate the influence of the Perme Score ^‡^ on ICU ^†^ length of stay in the postoperative period of cardiac surgery and investigate the association of preoperative variables with postoperative mobility.	- The Perme Score ^‡^ on days 2 and 3 was associated with ICU ^†^ length of stay: (β= -0.76; IC95% -1.19 to -0.33, *p* = 0.001) and (β= -2.67; IC95% -3.38 to -1.95, *p* <0.001), respectively.	Prospective longitudinal study	44
				- An increase of 4.6 points in the Perme Score ^‡^ reduced ICU ^†^ length of stay by one day, regardless of the type of surgical procedure;		
				- Preoperative pulmonary function was one of the main independent predictors of mobility status during the first three days of ICU ^†^ admission, as well as left ventricular ejection fraction and extracorporeal circulation time on day 1 ( *p* = 0.006), age and left ventricular ejection fraction on day 2 ( *p* = 0.002), and Maximum Expiratory Pressure on day 3 ( *p* < 0.001).		
Nawa, et al., 2022 ^( 35 )^	PLoS One	EUA*	To address variations in clinical characteristics, use of MV ^¶^ , and risk factors associated with mobility level during ICU ^†^ stay in critically ill patients with COVID-19.	- The overall rate of patients out of bed was 63.3%, and those able to walk 30 meters was 20.5% at ICU ^†^ discharge;	Retrospective cohort study	949
				- The percentage of patients who were able to get out of bed during ICU ^†^ admission was lower in patients on MV ^¶|^ (36.4% vs. 72%, *p* < 0.001), in the elderly (51.2% vs. 64.1%, *p* < 0.001) and in frail patients (41.2% vs. 56.5% in pre-frail vs. 65.7% in non-frail; *p* < 0.001);		
				- After adjusting for confounding factors, the independent predictors of improvement in mobility level were frailty (OR 0.52; 95%CI: 0.29-0.94; *p* = 0.03) and higher Perme Score ^‡^ at admission (OR 0.35; 95%CI 0.28-0.43, *p* < 0.001).		
Yen, et al., 2022 ^( 39 )^	NeuroRehabilitation	Taiwan	To investigate the effects of early progressive mobilization on functional mobility and the rate of out-of-bed mobility achieved by patients with moderate to severe traumatic brain injury.	- At ICU ^†^ discharge, IG ^‡‡‡^ patients (progressive early mobilization) were at Level 1 (9.5%), Level 2 (33.3%) and Level 3 (52.4%), while CG ^****^ patients remained at Level 0 on the Modified ICU ^†^ Mobility Scale;	Interventional study	86
				- Initial Perme Score ^‡^ in the CG ^****^ was 3.23±2.03 vs. 2.83±2.26 in the IG ^‡‡‡^ ;		
				- The IG ^‡‡‡^ showed a significant increase in the total Perme Score ^‡^ at ICU ^†^ discharge (IG ^‡‡‡^ 6.62±4.33 vs. CG ^****^ 3.64±1.66, *p* = 0.001, η2p= 0.995);		
				- At ICU ^†^ discharge, only the subscore for the level of assistance required for mobility on the Perme Score ^‡^ showed a difference (CG ^****^ 3.64±1.66 vs. IG ^‡‡‡^ 6.62±4.33, *p* = 0.001).		
Nascimento, et al., 2023 ^( 24 )^	Canadian Journal of Respiratory Therapy	Brazil	To assess the mobility of patients with COVID-19 using the Perme Score ^‡^ outside the ICU ^†^ setting and correlate the score value with the length of hospital stay.	- An average increase of 7.3 points (95%CI 5.7-8.8, *p* < 0.001) between admission to the IU ^**^ and hospital discharge;	Retrospective cohort study	69
				- Average Perme Score ^‡^ values on the IU ^**^ were 17.5 (15.8-19.3) and at hospital discharge were 24.8 (23.3-26.3);		
				- There was no association between the Perme Score ^‡^ values and length of hospital stay (0.929 95%CI; 0.861-1.002, *p* = 0.058);		
				- It was observed that 17.9% of patients achieved the maximum score on the Perme Score ^‡^ , of whom only one patient (1.4%) had the maximum score at both admission and discharge.		
Rittel, et al., 2023 ^( 43 )^	Dimensions of Critical Care Nursing	USA*	To assess mobility and self-care among elderly patients admitted to the ICU ^†^ and identify barriers to early intervention.	- Initial Perme Score ^‡^ (IQR ^‡‡^ 25-75) 23 (11.5-28) and final (IQR ^‡‡^ 25-75) 27 (16-31);	Retrospective cohort study	43
				- Of the total number of patients, 76% showed improvement in the Perme Score ^‡^ ;		
				- Of the patients with improvement in the Perme Score ^‡^ , the median was (IQR ^‡‡^ 25-75) 9.4 (3,1-15.6);		
				- Reasons for non-mobilization were: lack of staff or sufficient time 17%, mental inability to follow instructions 8%, being under active sedation 6%, being hemodynamically unstable 3%, and transition to comfort care/hospice 3%.		
Tavares, et al., 2023 ^( 25 )^	Heart & Lung - The Journal of Cardiopulmonary and Acute Care	Brazil	To evaluate strength, mobility, and ICU ^†^ acquired weakness among individuals with and without COVID-19 and determine the Perme Score ^‡^ cutoff for ICUAW ^§§§§^ .	- Perme Score ^‡^ at ICU ^†^ discharge, COVID-19 Group 18.1 (15.5-20.7) vs. non-COVID-19 Group 18.3 (15-21.5), *p* = 0.20;	Cross-sectional study	48
				- Perme Score ^‡^ at hospital discharge COVID-19 Group 27.2 (24.6-29.9) vs. non-COVID-19 Group 27.6 (24.4-30.8), *p* = 0.65;		
				- A one-unit increase in the Perme Score ^‡^ reduced the length of hospital stay by 1.04 days for other pathologies and 8.30 days for COVID-19;		
				- The cutoff point with the highest sensitivity (0.82) and specificity (0.70) for detecting ICUAW ^§§§§^ in the Perme Score ^‡^ was 18 points.		

*USA = United States of America; ^†^ICU = Intensive Care Unit; ^‡^Perme Score = Perme Intensive Care Unit Mobility Score; ^§^IMS = ICU Mobility Scale; ^||^MRC *=* Medical Research Council; ^¶^MV = Mechanical Ventilation; **IU = Inpatient Unit; ^††^FSS-ICU = Functional Status Score for the Intensive Care Unit; ^‡‡^IQR = Interquartile range; ^§§^APACHE = Acute Physiology and Chronic Health disease Classification System; ^||||^MRC-SS = Medical Research Council Sum Score; ^¶¶^PBM = Potential Barriers to Mobilization; ***MDC = Minimal Detectable Change; ^†††^CVA = Cerebrovascular Accident; ^‡‡‡^IG = Intervention Group; ^§§§^10mWT = 10 meter Walk Test; ^||||||^BBS = Berg Balance Scale; ****CG = Control Group; ^††††^HGS = Handgrip Strength; ^‡‡‡‡^ERBI = Early Rehabilitation Barthel Index; ^§§§§^ICUAW = ICU-Acquired Weakness

The studies addressed a variety of topics, such as description and reliability of the instrument designed by Christiane Strambi Perme^(16-17)^; translation and cultural adaptation into other languages^(5,18-19)^; association between functional mobility and clinical characteristics^(20)^; assessment of mobility and patient outcomes^(21-25,30)^; assessment of mobility following specific interventions, such as the early mobilization protocol^(26)^, comparison between conventional physiotherapy and cycle ergometer^(27-28)^; assessment of mobility and potential barriers to mobilization^(29-30)^ and the use of the Perme Score as a validation tool for other instruments^(31-33)^.

Most of the studies were conducted primarily in the ICU. Only two studies were carried out in hospital inpatient units (wards)^(24,36)^. Another relevant detail concerns the specific profiles of the patients monitored, which included cases of COVID-19^(24,34-35)^, liver transplantation^(36)^, cardiac surgery^(37)^, tracheostomized^(38)^ and organophosphate poisoning^(29)^.

## Discussion

The Perme Intensive Care Unit Mobility Score was developed to assess functional mobility and comprises the following subcategories: mental status, potential barriers to mobility, functional strength, bed mobility, transfers, gait and endurance. The instrument demonstrated an agreement of 94.29% (68.57%-100%) between evaluators^(16)^, with its reliability evaluated in a cardiovascular ICU in the same year^(17)^.

After assessing the reliability of the instrument, it was translated and validated into Brazilian Portuguese^(5)^, Spanish^(19)^ and German^(18)^, which, in addition to the translation and cross-cultural validation, obtained inter-rater reliability (physiotherapists and nurses) of 96% (93-97%), highlighting the role of the nursing professional in the context of functional mobility assessment^(18)^.

Regarding publications on the Perme Score, the first studies described the instrument. Later, studies focused on specific clinical conditions, postoperative complications and clinical outcomes. Higher Perme Score values were associated with hospital discharge to home^(21)^.

Different Perme Score values guide discharge referrals. Patients discharged to home had a Perme Score of 29; to home care, 12; to rehabilitation hospitals, 26; to specialized nursing services, 13; and for death, 7^(21)^. A similar discharge profile was observed in patients admitted to ICUs in the United States of America: home 26.05 (±5.42), long-term care institutions 18.65 (±8.43), specialized nursing services 17.38 (±7.72), and rehabilitation 20.3 (±7.48)^(41)^. In this context, the Perme Score proves to be a useful tool for assessing the effectiveness of in-hospital rehabilitation, guiding efforts for the appropriate referral of patients within the Healthcare Network (HCN) to enhance mobility and, consequently, achieve better outcomes.

The Perme Score for the death outcome was lower (0.57; ±1.98) when compared to the score at hospital discharge (7.27; ±8.16), *p*< 0.0001, and the use of vasoactive drugs and sedatives was higher in the death group, *p*≤ 0.0001. In addition, clinical hospitalizations had a lower Perme Score than surgical hospitalizations (4.15 ±7.30 vs. 5.44±6.75, *p*< 0.01)^(40)^. Considering that immobility is associated with a series of negative outcomes, mobility assessment, as well as the implementation of an early mobilization program, should be incorporated in care management.

Although most studies applied the Perme Score in the ICU, some utilized it in Inpatient Units (IU). An average increase in functional mobility was observed in the IU (7.3; 95% CI 5.7-8.8, *p*< 0.001) between admission and hospital discharge. The mean Perme Score values at IU admission were 17.5 (95% CI 15.8–19.3), and at hospital discharge 24.8 (95%CI 23.3-26.3)^(24)^. These studies extend the use of the Perme Score beyond the ICU, demonstrating the feasibility of applying the tool to manage mobility in different healthcare settings.

The use of early mobilization protocols to improve the functional mobility status of ICU patients contributes to a significant increase in the Perme Score from the first day of admission to the last day of rehabilitation^(26)^. Early ambulation interventions were also studied, showing that patients who ambulated early experienced a smaller decrease in the Perme Score after cardiac valve surgery^(37)^. Additionally, early mobilization demonstrated better outcomes in the functional mobility of patients with moderate to severe traumatic brain injury (TBI). The Intervention Group (IG) showed a significant increase in the Perme Score at ICU discharge compared to the Control Group (CG) (IG 6.62 ± 4.33 vs. CG 3.64 ± 1.66, *p*= 0.001)^(39)^.

In the same context, the use of a cycle ergometer in rehabilitation after acute cerebrovascular accident demonstrated greater mobility and functionality in relation to conventional physiotherapy^(27)^. Early mobilization should prioritize social reintegration to minimize or reverse the impacts of hospitalization through activities that promote independence. Therefore, early mobilization should be a goal for the entire multidisciplinary team.

Potential barriers to mobilization should be assessed by the multidisciplinary team and, once identified, strategies can be developed to minimize them. Higher scores on Potential Barriers to Mobilization were associated with higher Perme Score values, shorter length of stay on mechanical ventilation and shorter total length of stay in the ICU^(30)^.

A study conducted in the USA aimed to assess the mobility and self-care of elderly patients in the ICU and identify barriers to early mobilization. An initial Perme Score of 23 (IQR 11.5-28) and a final score of 27 (IQR 16-31) were observed, with 76% of patients showing improvement. The reasons for the lack of early mobilization included insufficient staff or time (17%), cognitive inability to follow instructions (8%), sedation (6%), hemodynamic instability (3%), and transition to comfort care/hospice (3%)^(43)^.

The Perme Score demonstrated predictive power in the postoperative period of liver transplantation, showing an inverse association between the score and the duration of mechanical ventilation (*p*= 0.042), as well as the number of physiotherapy interventions in the inpatient unit (*p*= 0.001)^(36)^.

Functional mobility performance was evidenced after the use of the speaking valve in tracheostomized individuals, with the Perme Score increasing from 11.3 (IQR 10.1-12.0) to 18.2 (IQR 16.2-20.1); *p*< 0.01. The benefits of the speaking valve are widely discussed in relation to speech processes and adjustments to more physiological respiratory patterns, as well as swallowing processes. However, the indirect benefits of the speaking valve are less frequently addressed. When initiated shortly after the cessation of mechanical ventilation in tracheostomized individuals, it can improve mobility capacity^(38)^.

The Perme Score was used as a comparative parameter in the translation and cross-cultural adaptation of the Functional Status Score for the Intensive Care Unit (FSS-ICU)^(31)^ and the ICU mobility scale *(IMS)* into Turkish^(32)^. The validation of the Perme Score for Turkish was not included in this review, as the study has not been published and is only available in the dissertation database of Muğla Sıtkı Koçman University , written in Turkish. In addition to these studies, the Perme Score was also employed in the translation of the Early Rehabilitation Index (ERI) into Brazilian Portuguese^(33)^. Due to its ease of application and consideration of potential mobility barriers, it has been used as a comparative index for new instruments.

It is important to note that no additional tests for the validation of psychometric properties and construct validity were found in the literature. Although not mandatory, it is highly recommended that, after the translation and adaptation process, researchers ensure that the new version demonstrates the necessary measurement properties for the intended application. This would provide greater confidence that the adapted instrument measures a construct comparable to the original^(44)^.

A limitation of this study is the absence of psychometric validation of the scale. Furthermore, the chosen method aims to map the literature in a specific field of interest rather than identify the best evidence for a health intervention. Therefore, it was not possible to classify the robustness of the evidence, only to track it and anticipate its potential. Despite these limitations, a strong point of this review is the comprehensive search strategy and the standardized data extraction process required by the JBI. Further studies are suggested to conduct additional tests of the version adapted to Brazilian Portuguese, facilitating the use of the scale in settings beyond the ICU, as has already been demonstrated.

As a contribution to scientific knowledge in the health field, the possibility of using the Perme Score in other hospital sectors stands out, assisting in the rapid and objective measurement of functional mobility, including considering extrinsic conditions to the patient.

## Conclusion

This scoping review mapped the application of the Perme Score in the description and assessment of the instrument’s reliability, as well as its use as a reference for the translation and cultural adaptation of other instruments. Additionally, it addressed the assessment of functional mobility in association with clinical characteristics, clinical outcomes, and potential barriers to mobility. The review also highlighted the use of the Perme Score in intervention protocols for early mobilization, the assessment of functional mobility during conventional physiotherapy, and the use of the cycle ergometer.

The different scores on the functional mobility scale were associated with clinical characteristics and their outcomes, interventions, and potential mobility barriers.

Although the Perme Score was initially developed for assessing functional mobility in ICU, it has demonstrated great potential for use in different contexts. It is an instrument that allows functional mobility to be measured quickly, objectively and specifically, while also considering factors external to the patient.

## Footnotes

1 USD exchange rate = R$ 5.84, on 02/01/2025
