# Supplementary material for: Evidence of the use of the Perme Intensive Care Unit Mobility Score in hospitalized adults: a scoping review
Source: Rev Lat Am Enfermagem. 2025 May 2;33:e4542. doi: 10.1590/1518-8345.7491.4542 (PMC12058116; doi:10.1590/1518-8345.7491.4542)
Supplement: Translation

# Translation: Evidências da utilização do Perme *Intensive Care Unit Mobility Score* em indivíduos adultos hospitalizados: revisão de escopo

**DOI:** 10.1590/1518-8345.7491.4543

**Authors:** Maria Helena Lenard, Clovis Cechinel, Tissiane Bona Zomer, João Alberto Martins Rodrigues, Maria Angélica Binotto, Rossana Spoladore

**Affiliations:** 1 Universidade Federal do Paraná, Curitiba, PR, Brasil; 2 Hospital Municipal Zilda Arns, Curitiba, PR, Brasil; 3 Bolsista do Ministério da Saúde, Secretaria de Gestão do Trabalho e da Educação na Saúde, Programa de Residência em Saúde do Idoso, Brasil; 4 Universidade Estadual do Centro-Oeste, Departamento de Educação Física, Irati, PR, Brasil

**Keywords:** Limitação da Mobilidade, Deambulação Precoce, Modalidades de Fisioterapia, Pacientes Internados, Enfermagem em Reabilitação, Revisão

## Abstract

mapear a literatura científica referente ao uso do Perme *Intensive Care Unit Mobility Score* em indivíduos adultos hospitalizados.

revisão de escopo, estruturada nas diretrizes metodológicas do *Joanna Briggs Institute - Evidence Synthesis Groups*, com buscas em sete bases de dados e literatura cinzenta. Os estudos foram selecionados por dois revisores, utilizando um instrumento para a extração dos dados.

a análise dos 29 estudos selecionados mostrou predomínio de estudos longitudinais (34,48%), realizados no Brasil (48,27%) em Unidades de Terapia Intensiva (29%), e publicados entre 2020 e 2021 (48,24%). Os estudos evidenciaram a utilização do Perme *Score* para descrição e confiabilidade do instrumento, tradução e adaptação cultural, associação entre mobilidade funcional, características clínicas e desfechos, avaliação da mobilidade após intervenções, avaliação da mobilidade e potenciais barreiras para a mobilização, e uso da pontuação para validação de outros instrumentos e perfis clínicos diversos.

o Perme *Score* é um instrumento que permite mensurar a mobilidade física, incluindo as possíveis barreiras à mobilidade, com potencial para utilização em cenários externos à Unidade de Terapia Intensiva, em estudos de intervenção para mobilização precoce e predição de desfechos da hospitalização.

## Destaques

**(1)** O Perme *Score* apresenta grande potencial de uso em diferentes cenários. **(2)** Estudos posicionam o enfermeiro no contexto da avaliação da mobilidade funcional. **(3)** O Perme *Score* tem sido empregado em protocolos de intervenção para mobilidade precoce.

## Introdução

A instituição hospitalar é considerada um espaço que proporciona alívio de sintomas, recuperação da saúde e acesso a diagnóstico. No entanto, dependendo dos cuidados e tratamentos fornecidos, assim como de outros diversos aspectos, a internação hospitalar pode se tornar um fator desestruturante, resultando em maior deterioração clínica e funcional, aumento do tempo de permanência, descompensação das multimorbidades e maior risco de mortalidade^(1)^.

As consequenciais do declínio de mobilidade durante a hospitalização podem se estender por até́ cinco anos após a alta, sendo decorrentes de internações prolongadas que se relacionam à idade, à gravidade da doença e ao tipo de admissão (aguda/eletiva). Essas foram as conclusões de um estudo de coorte realizado com 10.430 indivíduos, no grupo Unidade de Terapia Intensiva (UTI) (n= 5.215) e na enfermaria (n= 5.215), com mediana de idade de 60 anos (intervalo de 44 a 72) em Edinburgh (Reino Unido). Houve associação do grupo UTI com maior mortalidade (RR 1,33; IC95%, 1,22–1,46, *p*= 0,001), maior custo (USD 25.608 vs. USD 16.913/paciente ^1^) e maior tempo de hospitalização (51%)^(2)^.

Estima-se um descondicionamento físico de 30% associado à hospitalização^(3)^. Desse modo, o declínio funcional não está relacionado apenas ao quadro clínico que motivou a hospitalização, assim como a recuperação não é automática após a resolução do problema que a suscitou^(4)^. Destaca-se que a mobilidade funcional é um preditor de saúde. Assim, a avaliação das funções locomotoras no contexto hospitalar e a compreensão das barreiras à mobilização precoce tornam-se imprescindíveis.

A mobilização precoce deve ser entendida como parte do processo de reabilitação do paciente hospitalizado, principalmente na UTI, minimizando a fraqueza muscular e a piora da função física^(5)^. Há diversas escalas que avaliam aspectos funcionais na UTI, como o *Physical Function in Intensive care Test scored*, *Chelsea Critical Care Physical Assessment tool*, Perme *Intensive Care Unit Mobility Score*, *Surgical intensivecare unit Optimal Mobilization Score*, *ICU Mobility Scale* e *Functional Status Score for the ICU* ^(6)^. Nenhuma delas foi considerada “padrão-ouro” na quantificação da mobilidade funcional, pois deveriam ser de aplicação rápida e objetiva. No entanto, o Perme *Intensive Care Unit Mobility Score* considera as condições extrínsecas ao paciente que interferem em sua mobilidade no leito, tais como a presença de acessos, tubos e drenos torácicos, que podem ser interpretadas como barreiras à mobilidade. A presença desses dispositivos não é pontuada nem considerada na maioria das escalas.

O Perme *Intensive Care Unit Mobility Score*, idealizado por Christiane Strambi Perme e aqui designado de Perme *Score*, foi traduzido e adaptado para o português. Ele é composto por sete categorias que contemplam estado mental, potenciais barreiras à mobilidade, força funcional, mobilidade no leito, transferências, dispositivos de auxílio para deambulação e medidas de resistência. A pontuação varia de 0 a 32 pontos; uma pontuação mais alta indica maior mobilidade e menor necessidade de assistência, enquanto uma pontuação baixa indica menor mobilidade e maior necessidade de assistência^(7)^.

Apesar de ser um instrumento relativamente novo, já traduzido para diferentes idiomas, e trazer como inovação a avaliação das potenciais barreiras à mobilidade na UTI, é indispensável representar as publicações sobre o escore na literatura, considerando as diferentes condições clínicas e cenários. Após uma busca preliminar em bases públicas de registro de protocolos de revisões e bases de dados online, e diante da inexistência de revisões sistemáticas ou registros de protocolos sobre essa escala, considerou-se imperioso o desenvolvimento desta revisão, que teve como objetivo mapear a literatura científica referente ao uso do Perme *Intensive Care Unit Mobility Score* em indivíduos adultos hospitalizados.

## Método

### Tipo de estudo

Trata-se de uma revisão de escopo, um método sistemático que identifica e sintetiza o conhecimento por meio da literatura existente ou emergente, mapeia a extensão e o alcance do tema, a natureza da literatura e determina possíveis lacunas^(8)^. A revisão foi desenvolvida segundo as recomendações do *JBI – Evidence Synthesis Groups,* método recomendado para revisões de escopo^(9)^ *,* além das diretrizes preconizadas pelo *Preferred Reporting Items for Systematic Reviews and Meta-Analyses Extension for Scoping Reviews* (PRISMA-ScR), e foi registrada na *International Platform of Registered Systematic Review and Meta-analysis Protocols* (INPLASY), sob o número 2023100031 (https://inplasy.com/inplasy-2023-10-0031).

Desse modo, a revisão de escopo foi operacionalizada em cinco etapas: 1- estabelecimento da questão de pesquisa; 2- identificação de estudos relevantes; 3- seleção e inclusão de estudos; 4- organização dos dados; e 5- coleta, síntese e relato dos resultados^(10-11)^. Assim, elaborou-se a pergunta e os elementos principais de busca desta revisão utilizando a estratégia PCC (*P – Population or Patients; C – Concept; C – Context*)^(12)^, sendo P (indivíduos adultos), C (Perme *Intensive Care Unit Mobility Score*) e C (hospitalar). No Contexto, ambiente hospitalar refere-se a qualquer setor de atendimento dentro da unidade hospitalar.

Com isso, a seguinte questão foi formulada: quais são as evidências disponíveis na literatura sobre o uso do Perme *Intensive Care Unit Mobility Score* em indivíduos adultos hospitalizados?

### Critérios de seleção

Com relação à elegibilidade dos estudos, os critérios de inclusão foram: (1) serem pesquisas primárias e secundárias, empíricas, quantitativas e qualitativas, de qualquer desenho ou metodologia; (2) conterem as variáveis de interesse “Perme *Score*” e “hospitalização*“*; (3) estarem publicados em inglês, espanhol, português ou francês; e (4) terem sido publicados a partir de 2014, ano de publicação da escala. Os critérios de exclusão dos estudos foram: cartas ao editor, resumos em anais de eventos, dissertações, teses, monografias e relatos de caso que não apresentassem a variável de interesse da pesquisa.

### Busca e coleta de dados

Inicialmente, a estratégia de busca foi construída a partir da identificação dos descritores *Medical Subject Headings* (MeSH): *mobility limitation, early ambulation, physical therapy modalities* e *inpatients*, associados ao termo livre “Perme”. Em seguida, esses descritores foram traduzidos para os termos específicos de cada base pesquisada, como Descritores em Ciências da Saúde (DeCS) e *Embase Subject Headings* (Emtree).

Contudo, as bases de dados e os portais tenderam a zero quando os descritores foram associados ao operador booleano “AND”. Diante disso, buscou-se o auxílio de um profissional bibliotecário, a fim de minimizar a possibilidade de erros. Desse modo, a estratégia de busca foi redefinida para incluir apenas os termos livres: “Perme” OR “Perme *scale*” OR “Perme *score*”.

Em outubro de 2023, foram realizadas buscas nas seguintes bases de dados: Biblioteca Virtual em Saúde*,* EMBASE, PEDro, PubMed, SciELO, Scopus e *Web of Science,* e os títulos e resumos dos artigos foram encaminhados para dois revisores avaliarem a elegibilidade de modo independente. Para a literatura cinzenta, optou-se por realizar a busca no Catálogo de Teses e Dissertações da Coordenação de Aperfeiçoamento de Pessoal de Nível Superior (CAPES). Entre os estudos de literatura cinzenta que atenderam aos critérios de inclusão e exclusão, todos já possuíam artigos publicados em periódicos, os quais foram incluídos na revisão.

Os resultados das buscas textuais foram exportados e transferidos para o gerenciador de referências gratuito Mendeley^U+00AE^, uma ferramenta que permite acesso por múltiplos pesquisadores e organiza as referências em pastas separadas. As duplicatas foram removidas, mantendo-se apenas um dos títulos. Posteriormente, realizou-se a leitura dos títulos e resumos, com a exclusão de artigos que não atendiam aos critérios de inclusão ou que contivessem algum dos critérios de exclusão. Por fim, os textos selecionados foram disponibilizados aos revisores para leitura completa, finalizando o processo de inclusão.

Os pesquisadores, responsáveis pela revisão, são profissionais com expertise em gerontologia e membros de um Grupo Multiprofissional de Pesquisa sobre Idosos.

Cabe ressaltar que se utilizou o coeficiente de concordância Kappa para descrever a intensidade de concordância entre os revisores, a qual é baseada no número de respostas concordantes, ou seja, na frequência em que os resultados coincidem entre os revisores^(13)^. Neste estudo, a concordância bruta entre os revisores foi de 96,81%, o que corresponde a um coeficiente Kappa superior a 0,90. Além disso, um terceiro revisor avaliou divergências na seleção dos estudos para a tomada de decisão final sobre a inclusão ou exclusão, a fim de minimizar o risco de viés.

O número de artigos encontrados em cada base de dados e a soma de todas as bases foram registrados no fluxograma PRISMA^(14)^, assim como o processo de seleção e os motivos de exclusão.

### Extração e análise dos dados

A extração e a análise descritiva dos dados foram realizadas por meio de um roteiro elaborado pelos próprios autores, que abrange os critérios de elegibilidade previamente definidos, conforme amplamente recomendado pelo JBI*.* Neste sentido, incluiu-se: nome do autor e ano de publicação, periódico, país de origem, objetivos, resultados, desenho do estudo e tamanho da amostra.

Devido ao método escolhido, não houve necessidade de realizar uma avaliação formal quanto à qualidade metodológica dos estudos incluídos. Porém, a adesão aos itens de avaliação do *checklist* PRISMA *Extension for Scoping Reviews* (PRISMA-ScR) visou garantir o rigor metodológico do conteúdo^(15)^. Por fim, realizou-se a análise temática do conteúdo para identificar os pontos convergentes na literatura, delimitar os pontos fortes do assunto e as lacunas existentes.

### Aspectos éticos

Uma vez que os estudos utilizados são de domínio público, não houve necessidade de submissão ao Comitê de Ética em Pesquisa, conforme a Resolução CNS n° 510, de 2016.

## Resultados

A busca nos portais e bases de dados resultou em 1.873 estudos. Destes, 837 foram excluídos por serem duplicatas, utilizando o gerenciador de referências gratuito Mendeley^U+00AE^. Após a retirada das duplicatas, os 1.036 artigos selecionados para leitura de títulos e resumos foram organizados em uma planilha do Excel^U+00AE^ para continuidade das análises. Em seguida, 985 artigos foram excluídos após a leitura dos títulos, e 16 após a leitura dos resumos, resultando em 35 estudos para leitura na íntegra. Destes, excluíram-se seis, totalizando 29 estudos para revisão. Para minimizar possível risco de viés na seleção dos estudos, os revisores organizaram as referências no gerenciador de referências gratuito Mendeley^U+00AE^. O refinamento foi realizado por dois avaliadores independentes, buscando uma concordância de 100%. Um terceiro revisor avaliou as possíveis divergências ocorridas na seleção dos estudos para a tomada de decisão final sobre a inclusão ou exclusão dos mesmos. Tais procedimentos estão representados na Figura 1, no fluxograma do método *Preferred Reporting Items for Systematic Reviews and Meta-Analysis* (PRISMA), que ilustra a seleção dos artigos desta revisão^(14)^.

Na Figura 2, observa-se o delineamento metodológico, o foco do estudo, os locais de realização e os perfis clínicos, que são as principais informações encontradas.

**Figura 1 -:** Fluxograma PRISMA-ScR de seleção dos estudos. Curitiba, PR, Brasil, 2024

**Figura 2 -:** Representação esquemática dos achados dos estudos envolvendo o Perme *Intensive Care Unit Mobility Score*. Curitiba, PR, Brasil, 2024

Na Figura 3, observa-se a categorização dos artigos por autor/ano de publicação, periódico, país de origem, objetivos, resultados, desenho do estudo e tamanho da amostra.

Entre os estudos analisados, houve predomínio de publicações no ano de 2021 (n= 8; 27,58%), seguido por 2020 (n= 7; 24,14%), 2022 e 2023 (n= 3; 10,34% em cada ano), 2014, 2018 e 2019 (n= 2; 6,9% em cada ano) e, por fim, 2016 e 2017 (n= 1; 3,45% em cada ano).

Quanto aos periódicos, observou-se 25 diferentes, com prevalência do *Colombia Médica*, *NeuroRehabilitation*, *PLoS One* e Revista Brasileira de Terapia Intensiva (n= 2; 6,89% cada um). Os países que se destacaram como sede dos estudos foram: Brasil (n= 14; 48,27%), Estados Unidos da América (n= 6; 20,68%), Colômbia (n= 3; 10,35%), Índia e Turquia (n= 2; 6,9% cada um), Alemanha e Taiwan (n= 1; 3,45% cada um). Todos os artigos (n= 29) foram publicados em inglês.

Em relação ao delineamento metodológico, predominaram estudos longitudinais (n= 10; 34,48%). Os demais foram estudos metodológicos (n= 8; 27,58%), estudos de coorte retrospectivos (n= 4; 13,79%), estudos transversais (n= 3; 10,35%), estudos de intervenção (n= 2; 6,9%), ensaio clínico randomizado e ensaio clínico não randomizado (n= 1; 3,45% cada um). Observou-se um total de 3.399 participantes nos estudos, com tamanhos variando entre 18 e 949 participantes.

**Figura 3 –:** Caracterização dos estudos que compuseram a revisão de escopo (n = 29). Curitiba, PR, Brasil, 2024

Autor/ Ano	Periódico	País de origem	Objetivos	Resultados	Desenho do estudo	Tamanho da amostra
Perme, et al., 2014 ^( 16 )^	Methodist DeBakey Cardiovascular Journal	EUA*	Descrever uma ferramenta que avalia a mobilidade na UTI ^†^ e a confiabilidade, e abordar a aplicação clínica.	- O Perme *Score* ^‡^ é um instrumento de avaliação da mobilidade funcional em pacientes internados em UTI ^†^ , com alta confiabilidade e validade. A pontuação varia de 0 a 32 pontos, sendo que quanto maior a pontuação, maior a mobilidade e menor a necessidade de assistência.	Estudo metodológico	35
				- Concordância Kappa entre avaliadores: 94,29% (68,57%-100%). Os valores Kappa foram: item 2 (estado mental: paciente consegue seguir 2 entre 3 comandos), sem concordância (κ= 0); item 1(estado de alerta no contato inicial), concordância razoável (κ= 0,21-0,40); itens 5, 10, 12 e 13, concordância moderada (κ= 0,41-0,60); itens 4, 7, 9 e 11, concordância substancial (κ= 0,61-0,80); e itens 3, 6, 8, 14 e 15, alta concordância (κ= 0,81-1,00).		
Nawa, et al., 2014 ^( 17 )^	*Journal of Critical Care*	EUA*	Determinar a confiabilidade entre avaliadores do Perme *Score* ^‡^ em pacientes internados em UTI ^†^ Cardiovascular.	Os 15 itens do escore foram analisados isoladamente. A confiabilidade interavaliadores foi de: 1,0 para os itens 2, 3, 5, 6, 7, 8, 13 e 15; 0,82 para o item 1; 0,80 para o item 4; 0,60 para o item 9; 0,72 para os itens 10 e 11; 0,78 para o item 12; e 0,90 para o item 14. O coeficiente de correlação intraclasse foi de 0,98 (IC95% 0,97-0,99).	Estudo metodológico	20
Kawaguchi, et al., 2016 ^( 7 )^	Jornal Brasileiro de Pneumologia	Brasil	Realizar tradução, adaptação e validação transcultural do Perme *Score* ^‡^ e IMS ^§^ para o português do Brasil.	Os instrumentos apresentaram excelente concordância interobservadores (κ> 0,90) e confiabilidade (α> 0,90) em todos os domínios. Houve forte correlação positiva entre os dois instrumentos (r= 0,941; *p* < 0,001).	Estudo metodológico	103
Yang, et al., 2017 ^( 21 )^	*American Journal of Respiratory and Critical Care Medicine*	EUA*	Identificar o Perme *Score* ^‡^ dos pacientes na alta da UTI ^†^ e o destino final da alta.	A mediana do Perme *Score* ^‡^ para os pacientes de alta para casa foi de 29; *homecare* , 12; hospital de reabilitação, 26; centro de enfermagem especializado, 13; e óbito, 7. Escores mais elevados do Perme *Score* ^‡^ se associaram à alta para casa ( *p* < 0,05).	Estudo longitudinal prospectivo	157
Nydahl, et al., 2018 ^( 18 )^	*European Journal of Physiotherapy*	Alemanha	Traduzir o Perme *Score* ^‡^ para o idioma alemão e investigar a confiabilidade interavaliadores entre fisioterapeutas e enfermeiros.	A confiabilidade entre avaliadores (enfermeiros e fisioterapeutas) foi quase perfeita: 0,96 (IC95%: 0,93-0,97). Dois itens foram avaliados com índice de confiabilidade menor que 0,8: capacidade de seguir comandos (0,73, IC95%: 0,55-0,89) e dor (0,46, IC95%: 0,09-0,68). O tempo de preenchimento da escala foi de aproximadamente 30 segundos. Primeiro estudo que compara a aplicação entre diferentes profissionais.	Estudo metodológico	58
Wilches Luna, et al., 2018 ^( 19 )^	*Colombia M* é *dica*	Colômbia	Realizar tradução, adaptação cultural e confiabilidade interavaliadores das versões em espanhol do Perme *Score* ^‡^ e IMS ^§^ em pacientes de UTI ^†^ .	A confiabilidade interavaliadores da IMS ^§^ ficou entre 0,97 e 1,0, e para o Perme *Score* ^‡^ entre 0,99 e 1,0 nos dois momentos das medidas, até 24 horas da admissão e alta da UTI ^†^ .	Estudo metodológico	150
Moecke & Biscaro, 2019 ^( 22 )^	Fisioterapia Brasil	Brasil	Analisar o estado funcional de pacientes internados em UTI ^†^ e associar com os desfechos funcionais e clínicos e força muscular.	- Perme *Score* ^‡^ : 8,18±3,99 ao despertar da sedação e 13,68±6 na alta da UTI ^†^ ;	Estudo longitudinal prospectivo	40
				- O estado funcional e a fragilidade se associam a desfechos clínicos (alta/transferência e óbito) ( *p* = 0,022 e *p* = 0,019);		
				- A MRC ^||^ se associou à pontuação do Perme *Score* ^‡^ ao despertar ( *p* < 0,001) e alta da UTI ^†^ ( *p* = 0,002);		
				- Maiores pontuações do Perme *Score* ^‡^ se associam à MRC ^||^ > 48.		
Pereira, et al., 2019 ^( 36 )^	Revista Brasileira de Terapia Intensiva	Brasil	Avaliar o poder preditivo do Perme *Score* ^‡^ para complicações no pós-operatório de transplante hepático.	- Perme *Score* ^‡^ na admissão (5,5) e alta (23,6) da UTI ^†^ , na enfermaria 28,2±5, e na alta hospitalar 31,7±0,7;	Estudo observacional prospectivo	30
				- Ao comparar a avaliação inicial na enfermaria e na alta hospitalar, houve melhora do estado funcional, Perme *Score* ^‡^ 28,2 ± 5 a 31,7 ± 0,7, *p* < 0,001;		
				- O tempo de VM ^¶^ se associou ao Perme *Score* ^‡^ na alta da UTI ^†^ (r = -0,374; *p* = 0,042). Quanto maior o tempo de VM ^¶^ , menor o Perme *Score* ^‡^ na alta da UTI ^†^ ;		
				- O número de tratamentos fisioterapêuticos se associou inversamente ao Perme *Score* ^‡^ na UI ^**^ (r = -0,578; *p* = 0,001).		
Ozcan, et al., 2020 ^( 31 )^	*Disability and Rehabilitation*	Turquia	Traduzir e adaptar transculturalmente o instrumento FSS-ICU ^††^ para o turco e avaliar as propriedades psicométricas.	- A consistência interna foi alta (Cronbach’s α=0,949). A confiabilidade inter e intraavaliadores foi excelente (α= 0,955-0,996);	Estudo metodológico	50
				- O escore FSS-ICU ^††^ apresentou correlações moderadas a altas com outras medidas funcionais, como: Perme *Score* ^‡^ (Spearman’s r= 0,92), atividades de vida diária de Katz (r= 0,80) e força de preensão manual (r= 0,76–0,77).		
Cavalli, et al., 2020 ^( 40 )^	*Middle East Journal of Rehabilitation and Health Studies*	Brasil	Analisar os desfechos de alta ou óbito de pacientes internados em UTI ^†^ considerando idade, sexo, gravidade, internação, comorbidades, tempo de permanência na UTI ^†^ e no hospital.	- A média de idade foi de 54,91±19,7 anos, 62,5% do sexo masculino, com predomínio de afeções neurológicas (34%), em VM ^¶^ (63,9%), com permanência na UTI ^†^ de 5,76 dias, e Perme *Score* ^‡^ inicial de 4,48 ± 7,17. A pontuação inicial do Perme *Score* ^‡^ em casos de óbito foi menor (0,57±1,98) quando comparada à alta hospitalar (7,27±8,16), *p* < 0,0001. Além disso, os valores de uso de drogas vasoativas e sedativas foram maiores em casos de óbito, *p* ≤ 0,0001;	Estudo transversal	288
				- Houve diferença significativa do Perme *Score* ^‡^ inicial quanto ao tipo de internação: pacientes clínicos 4,15±7,30 e cirúrgicos 5,44±6,75, *p* < 0,01.		
Ceron, et al., 2020 ^( 38^ )	*Respiratory Care*	Brasil	Avaliar as alterações no desempenho da mobilidade com o uso de válvulas fonadoras em indivíduos traqueostomizados.	- O Perme *Score* ^‡^ aumentou de 11,3 (IQR ^‡‡^ 10,1-12,0) para 18,2 (IQR ^‡‡^ 16,2-20,1) imediatamente após o início do uso da válvula fonadora, *p* < 0,01.	Estudo de coorte prospectivo	18
				- Ao avaliar as categorias do Perme *Score* ^‡^ , observaram-se mudanças nas pontuações da “categoria de transferência” (“sentar para ficar de pé”, “equilíbrio estático em pé uma vez estabelecida a posição em pé” e “transferência da cama para a cadeira ou da cadeira para a cama”);		
				- O uso de válvulas fonadoras em traqueostomizados melhorou a mobilidade.		
Gatty, et al., 2020 ^( 26 )^	*Physiotherapy Theory and Practice*	Índia	Estudar a eficácia de um protocolo de mobilização precoce no estado de mobilidade de pacientes em UTI ^†^ .	- Perme *Score* ^‡^ no primeiro dia de UTI ^†^ , mediana (IQR ^‡‡^ ): grupo intervenção 6 (2,2-8) e grupo controle 6 (3-7); no primeiro dia de reabilitação, (IQR ^‡‡^ ): grupo intervenção 7 (5-9) e grupo controle 5 (3-8); e no último dia de reabilitação, (IQR ^‡‡^ ): grupo intervenção 18 (11-24,7) e grupo controle 7 (5-11);	Ensaio clínico não randomizado	63
				- A diferença na pontuação do Perme *Score* ^‡^ entre o primeiro e último dia de reabilitação foi de 9 (3,2-17) no grupo intervenção e 2 (0-5) no grupo controle, *p* ≤ 0,001;		
				- Houve um aumento significativo da mediana do Perme *Score* ^‡^ desde o primeiro dia de internação em UTI ^†^ até o último dia de reabilitação, sendo 12,5 (6,2-7,7) no grupo intervenção e 2 (0-6) no grupo controle, *p* < 0,001;		
				- O Perme *Score* ^‡^ foi comparado entre o primeiro dia de UTI ^†^ e o primeiro dia de reabilitação, e não se observou diferença no grupo intervenção ( *p* = 0,069) e no grupo controle ( *p* = 0,124).		
Lima, et al., 2020 ^( 23 )^	Fisioterapia em Movimento	Brasil	Determinar a relação entre a mobilidade funcional e os desfechos clínicos de pacientes internados em UTI ^†^ .	- Os desfechos clínicos (óbito/alta) se associaram aos domínios do Perme *Score* ^‡^ : estado mental ( *p* = 0,040), barreiras à mobilidade ( *p* = 0,016), força ( *p* = 0,01) e mobilidade no leito ( *p* = 0,024). A pontuação total do Perme *Score* ^‡^ foi 0 (0-10) no desfecho óbito e 10 (0-23) para alta, ( *p* = 0,002);	Estudo longitudinal prospectivo	33
				- O uso da VM ^¶^ esteve associado a baixas pontuações na escala, 2,40 ± 4,19, enquanto a ausência de seu uso a mais altas, 12,85±6,61, *p* = 0,000;		
				- Houve correlação positiva (R= 0,745) entre Perme *Score* ^‡^ e nível de sedação, e foi encontrada correlação inversa entre APACHE ^§§^ e Perme *Score* ^‡^ (R= -0,526), e APACHE ^§§^ e dias de VM ^¶^ (R= -0,602).		
Perme, et al., 2020 ^( 41 )^	*Journal of Acute Care Physical Therapy*	EUA*	Avaliar a associação entre os instrumentos Perme *Score* ^‡^ , MRC-SS ^||||^ e desfecho clínico de pacientes internados em UTI ^†^ .	- A média do Perme *Score* ^†^ foi de 23,56 (±7,09);	Estudo longitudinal prospectivo	250
				- O Perme *Score* ^†^ por destino de alta foi: domicílio 26,05±5,42, instituições de cuidado prolongado 18,65±8,43, serviços de enfermagem especializados 17,38±7,72, reabilitação 20,3±7,48, e outros 18±5,8;		
				- O Perme *Score* ^‡^ dos pacientes com alta foi diferente dos que receberam alta para reabilitação ( *p* < 0,001), serviço de enfermagem especializado ( *p* < 0,001), cuidados agudos de longo prazo ( *p* < 0,001), e cuidados paliativos/ *hospice* ou faleceram ( *p* < 0,001);		
				- Há correlação moderada entre o MRC-SS ^||||^ e o Perme *Score* ^‡^ (r= 0,66; *p* < 0,001);		
				- Os pacientes na UTI ^†^ com maior pontuação Perme *Score* ^‡^ ou MRC-SS ^||||^ no momento da avaliação do fisioterapeuta receberam alta para casa, enquanto aqueles com pontuações mais baixas necessitaram de cuidados pós-agudos;		
				- O Perme *Score* ^‡^ ou MRC-SS ^||||^ mais elevado indicou maior probabilidade de alta para casa.		
Pinto, et al., 2020 ^( 29 )^	*Critical Reviews™ in Physical and Rehabilitation Medicine*	Índia	Identificar PBM ^¶¶^ e avaliar a mudança na mobilidade durante permanência na UTI ^†^ em pacientes com intoxicação por organofosforados.	- O Perme *Score* ^‡^ (2,50–6,0; *p* ≤0,01) melhorou nos subcomponentes mobilidade no leito (1,83±0,35 para 7,23±3,84), transferências (0,37±0,02 para 4,78±3,84) e marcha (1,83±0,35 para 5,23±1,27) do dia 2 ao dia 10 de internação hospitalar;	Estudo transversal	37
				- O tubo endotraqueal e a infusão contínua de medicamentos foram identificados como as PBM ^¶¶^ (66,6%) pelo Perme *Score* ^‡^ .		
Wilches Luna, et al., 2021 ^( 42 )^	*Physiotherapy Research International*	Colômbia	Determinar a AMD ^***^ e a responsividade do Perme *Score* ^‡^ em adultos internados em UTI ^†^ .	- O valor da AMD ^***^ para o Perme *Score* ^‡^ foi de 1,36, demonstrando que os adultos internados em UTI ^†^ que tiveram diferença entre a primeira e a segunda superior a 1,36 pontos apresentaram diferença mínima detectável;	Estudo longitudinal prospectivo	142
				- A AMD ^***^ ocorreu em 80% dos pacientes;		
				- Houve diferença significativa no tempo de VM ^¶^ (0,011) e no tempo de permanência na UTI ^†^ ( *p* < 0,04).		
Cordeiro, et al., 2021 ^( 37 )^	*Journal of Clinical and Translational Research*	Brasil	Avaliar a associação entre deambulação precoce e funcionalidade em pacientes submetidos à cirurgia de troca valvar cardíaca.	- O grupo que caminhava teve um decréscimo de 11±2 no Perme *Score* ^‡^ , enquanto o grupo que não caminhava, um decréscimo de 13±2, *p* =0,34.	Estudo de coorte prospectivo	170
Da Rosa, et al., 2021 ^( 27 )^	*NeuroRehabilitation*	Brasil	Verificar a associação entre treinamento aeróbico de ciclismo e força muscular de membros inferiores, velocidade de marcha, equilíbrio, mobilidade e funcionalidade de indivíduos com AVE ^†††^ .	- Intervenção: treinamento aeróbico de ciclismo;	Ensaio clínico randomizado	20
				- Na análise da força muscular de membros inferiores houve melhora intergrupos entre pré e pós-intervenção. Houve melhora significativa em todos os grupos musculares, incluído o lado parético e não parético, apenas no GI ^‡‡‡^ ;		
				- No TC10m ^§§§^ e na EEB ^||||||^ houve diferença intragrupo no GI ^‡‡‡^ , *p* <0,001, e diferença intergrupo com melhor resultado para o GI ^‡‡‡^ , *p* < 0,001. Observou-se que o GI ^‡‡‡^ apresentou melhora no equilíbrio (pré 0±0, pós 28,9±7,45) e na velocidade da marcha (pré 0±0, pós 0,67±0,78);		
				- Em relação ao Perme *Score* ^‡^ , houve diferença entre os resultados do GI ^‡‡‡^ (pré 14,3±3,10 e pós 27,3±2,91, *p* < 0,001) e do GC ^****^ (pré 9,40±2,31 e pós 12,30±3,40, *p* < 0,001).		
				- Houve maior mobilidade em indivíduos após AVE ^†††^ agudo submetidos ao cicloergômetro.		
Luna, Perme e Gastaldi, 2021 ^( 30 )^	*Canadian Journal of Respiratory Therapy*	Colômbia	Identificar as PBM ^¶¶^ precoces em adultos através do Perme *Score* ^‡^ e associação com os dias em VM ^¶^ e o tempo de permanência na UTI ^†^ .	- Houve correlações inversas entre o total de dias em VM ^¶^ e o escore total de PBM ^¶¶^ na admissão (r= -0,773; *p* < 0,05) e na alta (r = -0,559; *p* < 0,05) da UTI ^†^ , assim como entre o tempo de internação na UTI ^†^ e o escore total de PBM ^¶¶^ na admissão (r = -0,420; *p* < 0,05) e na alta (r= 0,283; *p* < 0,05) da UTI ^†^ ;	Estudo observacional prospectivo	142
				- Houve correlação entre a pontuação total do item barreiras e a pontuação total do Perme *Score* ^‡^ (r= -0,91; *p* < 0,01).		
Özsoy, et al., 2021 ^( 32 )^	*Turkish Journal of Medical Sciences*	Turquia	Realizar a tradução e a adaptação cultural da IMS ^§^ para o turco e pesquisar as propriedades psicométricas.	- A confiabilidade inter e intraavaliadores da IMS ^§^ foi excelente, índice de Kappa de 0,87 (0,80-0,93) e 0,92 (0,87-0,96);	Estudo metodológico	70
				- Há correlações significativas da escala IMS ^§^ com a FSS-ICU ^††^ , Perme *Score* ^‡^ , Katz, FPM ^††††^ direita e esquerda (rs≥ 0,60; *p* < 0,05);		
				- Ambiente de UTI ^†^ , média de idade 69,65±10,73, 60% do sexo masculino, diagnóstico principal na admissão foi síndrome coronariana aguda 54,3%, Perme *Score* ^‡^ 21,32±5,09.		
Souza, et al., 2021 ^( 28 )^	Revista Pesquisa em Fisioterapia	Brasil	Avaliar a oferta energética e proteica de pacientes críticos submetidos à fisioterapia convencional associada ao cicloergômetro ativo (GI ^‡‡‡^ ) ou à fisioterapia convencional (GC ^****^ ) e correlacionar com MRC ^||^ , dados antropométricos e Perme *Score* ^‡^ .	- O nível de adequação calórica e proteica nos dois grupos foi de 73,9% e 69,5%, respectivamente. No GC ^****^ , a adequação calórica foi de 66,6% e de proteínas de 58,3%, enquanto no GI ^‡‡‡^ a adequação foi de 81,8% tanto calórica quanto proteica;	Estudo de intervenção	23
				- Houve redução significativa da circunferência da panturrilha no 9º dia em relação à inicial, *p* = 0,001. Não houve diferença no GC ^****^ e no GI ^‡‡‡^ , p= 0,053;		
				- Houve redução significativa da circunferência do braço no 9º dia em relação à inicial nos dois grupos (GC ^****^ *p* = 0,038 e GI ^‡‡‡^ *p* = 0,041);		
				- Houve correlação moderada e inversa entre o débito energético e o Perme *Score* ^‡^ final no GC ^****^ (r= 0,59; *p* = 0,03).		
Timenetsky, et al., 2021 ^( 34 )^	*PLoS One*	Brasil	Descrever o nível de mobilidade de pacientes com COVID-19 internados na UTI ^†^ e abordar fatores associados ao nível de mobilidade no momento da alta da UTI ^†^ .	- O Perme *Score* ^‡^ melhorou ao comparar a alta 20,0 (7-28) à admissão 7,0 (0-16) em UTI ^†^ , *p* < 0,001;	Estudo de coorte retrospectivo	136
				- Houve melhora de mobilidade durante a internação na UTI ^†^ em 64,7% dos pacientes, e a mediana do Perme *Score* ^‡^ foi de 1,5 (0,6-3,4);		
				- O grupo melhorado teve menor duração de VM ^¶^ : 10 (5-14) vs. 15 (8-24) dias, *p* = 0,021; menor tempo de internação: 25 (12-37) vs. 30 (11-48) dias, p< 0,001; e menor mortalidade em UTI ^†^ e hospitalar;		
				- Os preditores independentes para mobilidade foram menor idade, menor Índice de Comorbidade de Charlson, e não ter recebido terapia renal substitutiva.		
Reis, et al., 2021 ^( 33 )^	Revista Brasileira de Terapia Intensiva	Brasil	Traduzir e adaptar transculturalmente para o português do Brasil o *Early Rehabilitation Index* e verificar as propriedades psicométricas da ferramenta (ERBI ^‡‡‡‡^ ) na alta da UTI ^†^ .	- O ERBI ^‡‡‡‡^ teve confiabilidade adequada, com coeficiente alfa de Cronbach de 0,65;	Estudo metodológico	122
				- A confiabilidade entre avaliadores foi excelente, com coeficiente de 0,94 (IC95% 0,92-0,96);		
				- Observou-se a validade do ERBI ^‡‡‡‡^ por meio das correlações fortes e significativas com o escore total do Perme *Score* ^‡^ (rô= 0,72);		
				- Características da UTI ^†^ : Perme *Score* ^‡^ 25,5 (15-30), média de idade de 56 anos (46,8-66), sexo masculino 51%, motivo de internação (23% sepse, 19% pós operatório eletivo, 14% distúrbio cardiovascular), 68% VM ^¶^ , tempo de VM ^¶^ de 5 (3-8) dias, e alta hospitalar 88%.		
Nawa, et al., 2022 ^( 20 )^	*Colombia M* é *dica*	Brasil	Avaliar a influência do Perme *Score* ^‡^ no tempo de permanência em UTI ^†^ no pós-operatório de cirurgia cardíaca e investigar a associação das variáveis pré-operatórias com a mobilidade pós cirúrgica.	- O Perme *Score* ^‡^ nos dias 2 e 3 foi associado ao tempo de permanência na UTI ^†^ : (β= -0,76; IC95% -1,19 a -0,33, *p* = 0,001) e (β= -2,67; IC95% -3,38 a -1,95, *p* < 0,001), respectivamente.	Estudo longitudinal prospectivo	44
				- Aumento de 4,6 pontos no Perme *Score* ^‡^ reduziu em um dia o tempo de internação na UTI ^†^ , independente do procedimento cirúrgico;		
				- A função pulmonar pré-operatória foi um dos principais preditores independentes do estado de mobilidade nos primeiros três dias de internação na UTI ^†^ , assim como a fração de ejeção do ventrículo esquerdo e do tempo de circulação extracorpórea no dia 1 ( *p* = 0,006), idade e fração de ejeção do ventrículo esquerdo no dia 2 ( *p* = 0,002), e Pressão Expiratória Máxima no dia 3 ( *p* < 0,001).		
Nawa, et al., 2022 ^( 35 )^	*PLoS One*	EUA*	Abordar variações nas características clínicas, uso de VM ^¶^ , e fatores de risco associados ao nível de mobilidade durante a permanência na UTI ^†^ de pacientes gravemente enfermos com COVID-19.	- A taxa geral de pacientes fora do leito foi de 63,3%, e capazes de caminhar 30 metros de 20,5% na alta da UTI ^†^ ;	Estudo de coorte retrospectivo	949
				- A porcentagem de pacientes que conseguiram sair do leito durante a internação na UTI ^†^ foi menor nos pacientes em VM ^¶|^ (36,4% vs. 72%, *p* < 0,001), nos mais idosos (51,2% vs. 64,1%, *p* < 0,001) e em pacientes frágeis (41,2% vs. 56,5% em pré-frágeis vs. 65,7% em não frágeis; *p* < 0,001);		
				- Após ajuste para fatores de confusão, os preditores independentes de melhora do nível de mobilidade foram fragilidade (OR 0,52; IC95%: 0,29-0,94; *p* = 0,03) e maior Perme *Score* ^‡^ na admissão (OR 0,35; IC95% 0,28-0,43, *p* < 0,001).		
Yen, et al., 2022 ^( 39 )^	*NeuroRehabilitation*	Taiwan	Investigar os efeitos da mobilização precoce progressiva na mobilidade funcional e na taxa de mobilidade fora do leito alcançada por pacientes com traumatismo crânio encefálico moderado a grave.	- Na alta da UTI ^†^ , os pacientes do GI ^‡‡‡^ (mobilização precoce progressiva) estavam no Nível 1 (9,5%), Nível 2 (33,3%) e Nível 3 (52,4%), enquanto os pacientes do GC ^****^ permaneceram no Nível 0 na Escala Modificada de Mobilidade na UTI ^†^ ;	Estudo de intervenção	86
				- Perme *Score* ^‡^ inicial no GC ^****^ 3,23±2,03 vs. 2,83±2,26 no GI ^‡‡‡^ ;		
				- O GI ^‡‡‡^ teve um aumento significativo no Perme *Score* ^‡^ total no momento de alta da UTI ^†^ (GI ^‡‡‡^ 6,62±4,33 vs. GC ^****^ 3,64±1,66, *p* = 0,001, η2p= 0,995);		
				- Na alta da UTI ^†^ , apenas o subescore do nível de assistência necessária para a mobilidade do Perme *Score* ^‡^ apresentava diferença (GC ^****^ 3,64±1,66 vs. GI ^‡‡‡^ 6,62±4,33, *p* = 0,001).		
Nascimento, et al., 2023 ^( 24 )^	*Canadian Journal of Respiratory Therapy*	Brasil	Avaliar a mobilidade de pacientes com COVID-19 através do Perme *Score* ^‡^ fora do ambiente de UTI ^†^ e correlacionar o valor do escore com o tempo de internação.	- Aumento médio de 7,3 pontos (IC95% 5,7-8,8, *p* < 0,001) entre os momentos de admissão na UI ^**^ e a alta hospitalar;	Estudo de coorte retrospectivo	69
				- Valores médios do Perme *Score* ^‡^ na admissão na UI ^**^ foi de 17,5 (15,8-19,3) e na alta hospitalar de 24,8 (23,3-26,3);		
				- Não houve associação entre os valores do Perme *Score* ^‡^ e o tempo de internação (0,929 IC95%; 0,861-1,002, *p* = 0,058);		
				- Observou-se que 17,9% dos pacientes obtiveram pontuação máxima no Perme *Score* ^‡^ , dos quais apenas um (1,4%) paciente teve pontuação máxima na entrada e na saída.		
Rittel, et al., 2023 ^( 43 )^	*Dimensions of Critical Care Nursing*	EUA*	Avaliar a mobilidade e o autocuidado entre idosos internados em UTI ^†^ e identificar entraves para a intervenção precoce.	- Perme *Score* ^‡^ inicial (IQR ^‡‡^ 25-75) 23 (11,5-28) e final (IQR ^‡‡^ 25-75) 27 (16-31);	Estudo de coorte retrospectivo	43
				- Do total de pacientes, 76% apresentaram melhora do Perme *Score* ^‡^ ;		
				- Dos pacientes com melhora do Perme *Score* ^‡^ , a mediana foi (IQR ^‡‡^ 25-75) 9,4 (3,1-15,6);		
				- As razões para não mobilização foram: falta de pessoal ou de tempo suficiente 17%, incapacidade mental de seguir instruções 8%, estar sob sedação ativa 6%, ser hemodinamicamente instável 3%, e transição para cuidados de conforto/ *hospice* 3%.		
Tavares, et al., 2023 ^( 25 )^	*Heart & Lung - The Journal of Cardiopulmonary and Acute Care*	Brasil	Avaliar força, mobilidade e fraqueza adquirida em UTI ^†^ entre indivíduos com e sem COVID-19 e determinar o ponto de corte do Perme *Score* ^‡^ para ICUAW ^§§§§^ .	- Perme *Score* ^‡^ na alta UTI ^†^ , Grupo COVID-19 18,1 (15,5-20,7) vs. Grupo não COVID-19 18,3 (15-21,5), *p* = 0,20;	Estudo transversal	48
				- Perme *Score* ^‡^ na alta hospitalar Grupo COVID-19 27,2 (24,6-29,9) vs. Grupo não COVID-19 27,6 (24,4-30.8), *p* = 0,65;		
				- O aumento de uma unidade no Perme *Score* ^‡^ reduziu o tempo de internação em 1,04 dias em outras patologias e 8,30 dias para COVID-19;		
				- O ponto de corte com maior sensibilidade (0,82) e especificidade (0,70) para detecção da ICUAW ^§§§§^ no Perme *Score* ^‡^ foi de 18 pontos.		

*EUA = Estados Unidos da América; ^†^UTI = Unidade de Terapia Intensiva; ^‡^Perme *Score* = Perme *Intensive Care Unit Mobility Score*; ^§^IMS = *ICU Mobility Scale*; ^||^MRC *= Medical Research Council*; ^¶^VM = Ventilação Mecânica; **UI = Unidade de Internação; ^††^FSS-ICU = *Functional Status Score for the Intensive Care Unit*; ^‡‡^IQR = *Interquartile range*; ^§§^APACHE = *Acute Physiology and Chronic Health disease Classification System*; ^||||^MRC-SS = *Medical Research Council Sum Score*; ^¶¶^PBM = Potenciais Barreiras para a Mobilização; ***AMD = Alteração Mínima Detectável; ^†††^AVE = Acidente Vascular Encefálico; ^‡‡‡^GI = Grupo Intervenção; ^§§§^TC10m = Teste de Caminhada de 10 metros; ^||||||^EEB = Escala de Equilíbrio de Berg; ****GC = Grupo Controle; ^††††^FPM = Força de Preensão Manual; ^‡‡‡‡^ERBI = *Early Rehabilitation Barthel Index*; ^§§§§^ICUAW = Fraqueza Adquirida em UTI

Os estudos abordaram temas variados, como descrição e confiabilidade do instrumento idealizado por Christiane Strambi Perme^(16-17)^; tradução e adaptação cultural para outros idiomas^(5,18-19)^; associação entre a mobilidade funcional e as características clínicas^(20)^; avaliação da mobilidade e o desfecho dos pacientes^(21-25,30)^; avaliação da mobilidade após intervenções específicas, como o protocolo de mobilização precoce^(26)^, comparação entre fisioterapia convencional e cicloergômetro^(27-28)^; avaliação da mobilidade e potenciais barreiras para a mobilização^(29-30)^; e uso do Perme *Score* como ferramenta de validação de outros instrumentos^(31-33)^.

A maioria dos estudos teve como cenário principal a UTI. Apenas dois estudos foram desenvolvidos nas unidades de internação hospitalar (enfermaria)^(24,36)^. Outra informação relevante diz respeito aos perfis específicos dos pacientes acompanhados, que englobaram COVID-19^(24,34-35)^, transplante hepático^(36)^, cirurgia cardíaca^(37)^, pacientes traqueostomizados^(38)^ e intoxicados por organofosforados^(29)^.

## Discussão

O instrumento Perme *Intensive Care Unit Mobility Score* foi desenvolvido para avaliação da mobilidade funcional e é composto por subcategorias: estado mental, potenciais barreiras à mobilidade, força funcional, mobilidade no leito, transferências, marcha e resistência. O instrumento apresentou uma concordância de 94,29% (68,57%-100%) entre os avaliadores^(16)^, com sua confiabilidade avaliada em uma UTI cardiovascular no mesmo ano^(17)^.

Após a avaliação da confiabilidade do instrumento, ele foi traduzido e validado para o português do Brasil^(5)^, para o espanhol^(19)^ e para o alemão^(18)^, que além da tradução e validação transcultural obteve confiabilidade entre avaliadores (de fisioterapeutas e enfermeiros) de 96% (93-97%), destacando o papel do profissional enfermeiro no cenário da avaliação da mobilidade funcional^(18)^.

Em relação às publicações sobre o Perme *Score*, os primeiros estudos descreveram a pontuação do Perme *Score*. Posteriormente, surgiram estudos voltados para condições clínicas específicas, complicações pós-operatórias e desfechos clínicos. Valores mais elevados no Perme *Score* foram associados à alta hospitalar para casa^(21)^.

Diferentes valores do Perme *Score* orientam os encaminhamentos na alta hospitalar. Pacientes com alta para domicílio apresentaram um Perme *Score* de 29; para *homecare,* 12; para hospital de reabilitação, 26; para serviços de enfermagem especializados 13; e para óbito, 7^(21)^. Um perfil bem semelhante de destinos foi observado em pacientes internados em UTIs dos Estados Unidos da América: domicílio 26,05 (±5,42), instituições de cuidado prolongado 18,65 (±8,43), serviços de enfermagem especializados 17,38 (±7,72), e reabilitação 20,3±7,48^(41)^. Em vista disso, o Perme *Score* demonstra ser uma ferramenta útil para avaliação da efetividade da reabilitação intra-hospitalar, direcionando os esforços para o encaminhamento adequado do paciente na Rede de Atenção à Saúde (RAS), a fim de ampliar a mobilidade e, consequentemente, alcançar melhores desfechos.

A pontuação do Perme *Score* no desfecho óbito foi menor (0,57; ±1,98) quando comparada à pontuação na alta hospitalar (7,27; ±8,16), *p*< 0,0001, e foram superiores os valores de uso de drogas vasoativas e sedativos no grupo óbito, *p*≤ 0,0001. Além disso, internações clínicas apresentaram Perme *Score* inferior às cirúrgicas (4,15 ± 7,30 vs. 5,44±6,75, *p*< 0,01)^(40)^. Considerando que a imobilidade está associada a uma série de desfechos negativos, a avaliação de mobilidade, assim como a implementação de um programa de mobilização precoce, deve ser instituída no gerenciamento do cuidado.

Embora a maioria dos estudos tenha aplicado o Perme *Score* na UTI, alguns o utilizaram em Unidades de Internação (UI). Observou-se um aumento médio da mobilidade funcional nas UI (7,3; IC95% 5,7-8,8, *p*< 0,001) entre os momentos de admissão e alta hospitalar. Os valores médios do Perme *Score* na admissão na UI foram de 17,5 (IC95% 15,8-19,3), e na alta hospitalar de 24,8 (IC95% 23,3-26,3)^(24)^. Esses estudos ampliam a utilização do Perme *Score* para além da UTI, demonstrando a viabilidade da aplicação da ferramenta no gerenciamento da mobilidade em diferentes cenários de atenção à saúde.

O uso de protocolos de mobilização precoce no estado de mobilidade funcional dos pacientes na UTI contribui para o aumento significativo do Perme *Score* desde o primeiro dia de internação até o último dia de reabilitação^(26)^. A intervenção deambulação precoce também foi objeto de estudo, no qual os pacientes que deambulavam precocemente apresentaram menor decréscimo no Perme *Score* após cirurgia de válvula cardíaca^(37)^. Além disso, a mobilização precoce demonstrou melhores resultados na mobilidade funcional de pacientes com traumatismo cranioencefálico (TCE) moderado a grave. O Grupo Intervenção (GI) teve um aumento significativo no Perme *Score* na alta da UTI em comparação ao Grupo Controle (GC) (GI 6,62 ±4,33 vs. GC 3,64 ±1,66, *p*= 0,001)^(39)^.

Nesse mesmo sentido, a utilização do cicloergômetro na reabilitação após acidente vascular encefálico agudo demonstrou maior mobilidade e funcionalidade em relação à fisioterapia convencional^(27)^. A mobilização precoce deve ter como princípio a reinserção social, a fim de que os impactos da hospitalização sejam minimizados ou revertidos, através da realização de atividades que promovam a independência. Diante disso, a mobilização precoce deve ser uma meta de toda a equipe multidisciplinar.

As potenciais barreiras para a mobilização devem ser avaliadas pela equipe multidisciplinar e, uma vez identificadas, é possível promover estratégias para minimizá-las. Pontuações mais altas nas Potenciais Barreiras para a Mobilização associaram-se a maiores valores do Perme *Score*, menor tempo de permanência em ventilação mecânica e menor tempo total de permanência na UTI^(30)^.

Um estudo realizado nos EUA teve como objetivo avaliar a mobilidade e o autocuidado de idosos na UTI, além de identificar barreiras para a mobilização precoce. Observou-se um Perme *Score* inicial de 23 (IQR 11,5-28) e final de 27 (IQR 16-31), com 76% dos pacientes apresentando melhora. As razões para a não mobilização precoce incluíram falta de pessoal ou de tempo suficiente (17%), incapacidade cognitiva para seguir instruções (8%), sedação (6%), instabilidade hemodinâmica (3%) e transição para cuidados de conforto/*hospice* (3%)^(43)^.

O Perme *Score* apresentou poder preditivo no pós-operatório de transplante hepático, com uma associação inversa entre a pontuação e o tempo de ventilação mecânica (*p*= 0,042), bem como com o número de intervenções fisioterapêuticas na unidade de internação (*p*= 0,001)^(36)^.

O desempenho da mobilidade funcional foi evidenciado após o uso da válvula fonadora em indivíduos traqueostomizados, com aumento do Perme *Score* de 11,3 (IQR 10,1-12,0) para 18,2 (IQR 16,2-20,1); *p*< 0,01. Muito se discute sobre os benefícios da válvula fonadora no processo de fala e nos ajustes dos padrões respiratórios mais próximos do fisiológico, assim como no processo de deglutição. No entanto, pouco se aborda sobre os benefícios indiretos da válvula fonadora que, quando iniciada logo após a cessação da ventilação mecânica em indivíduos traqueostomizados, pode melhorar a capacidade de mobilidade^(38)^.

O Perme *Score* foi empregado como comparativo na tradução e adaptação transcultural do *Functional Status Score for the Intensive Care Unit* (FSS-ICU)^(31)^ e da *ICU mobility scale (IMS)* para o turco^(32)^. A validação do Perme *Score* para o turco não foi incluída nesta revisão, uma vez que tal trabalho não foi publicado, estando disponível apenas na base de dissertações da *Muğla Sıtkı Koçman University, no idioma turco.* Além desses estudos, o Perme *Score* também foi utilizado na tradução do *Early Rehabilitation Index* (ERI) para o português do Brasil^(33)^. Por ser um instrumento de fácil aplicação, que considera as potenciais barreiras à mobilidade, tem sido utilizado como índice de comparação para novos instrumentos.

É importante salientar que não foram encontrados na literatura testes adicionais para validação das propriedades psicométricas e validade do construto. Apesar de não ser obrigatório, é altamente recomendável que, após o processo de tradução e adaptação, os pesquisadores garantam que a nova versão demonstre as propriedades de medição necessárias para a aplicação pretendida. Dessa forma, haveria maior confiança de que o instrumento adaptado mede um construto comparável ao original^(44)^.

Como limitação do estudo, pode-se destacar a ausência de validação psicométrica da escala. Além disso, o método escolhido busca mapear a literatura em determinado campo de interesse, não tendo como objetivo encontrar a melhor evidência de uma intervenção em saúde. Sendo assim, não foi possível classificar a robustez da evidência, apenas rastreá-la e antecipar suas potencialidades. Apesar dessas limitações, esta revisão possui como ponto de destaque a estratégia de busca abrangente e o processo padronizado de extração de dados requerido pelo JBI. Sugere-se a realização de outros estudos, com a finalidade de conduzir testes adicionais da versão adaptada para o português do Brasil, propiciando a utilização da escala em outros ambientes, além da UTI, como já demonstrado.

Como contribuição para o conhecimento científico na área de saúde, destaca-se a possibilidade de utilização do Perme *Score* em outros setores hospitalares, auxiliando na mensuração da mobilidade funcional de forma rápida e objetiva, inclusive considerando condições extrínsecas ao paciente.

## Conclusão

A presente revisão de escopo mapeou a aplicação do Perme *Score* na descrição e avaliação da confiabilidade do instrumento, bem como sua utilização como padrão na tradução e adaptação cultural de outros instrumentos. Além disso, abordou a avaliação da mobilidade funcional relacionada às características clínicas, desfechos clínicos e potenciais barreiras para a mobilização. Também foi destacada a utilização do Perme *Score* em protocolos de intervenção para mobilização precoce, na avaliação da mobilidade funcional na fisioterapia convencional, e no uso do cicloergômetro.

As diferentes pontuações no escore de mobilidade funcional associaram-se a características clínicas e seus desfechos, intervenções e potenciais barreiras à mobilidade.

Apesar de o Perme *Score* ter sido um instrumento inicialmente desenvolvido para a avaliação da mobilidade funcional em UTI, ele mostrou grande potencial de uso em diferentes cenários. Trata-se de um instrumento que permite mensurar a mobilidade funcional de forma rápida, objetiva e específica, além de considerar condições extrínsecas ao paciente.

## Footnotes

1 Cotação do Dólar Estadunidense = R$ 5,84, em 01/02/2025.

# Translation: Evidencias de la utilización del Perme *Intensive Care Unit Mobility Score* en individuos adultos hospitalizados: revisión de alcance

**DOI:** 10.1590/1518-8345.7491.4541

**Authors:** Maria Helena Lenard, Clovis Cechinel, Tissiane Bona Zomer, João Alberto Martins Rodrigues, Maria Angélica Binotto, Rossana Spoladore

**Affiliations:** 1 Universidade Federal do Paraná, Curitiba, PR, Brasil; 2 Hospital Municipal Zilda Arns, Curitiba, PR, Brasil; 3 Becaria del Ministério da Saúde, Secretaria de Gestão do Trabalho e da Educação na Saúde, Programa de Residência em Saúde do Idoso, Brasil; 4 Universidade Estadual do Centro-Oeste, Departamento de Educação Física, Irati, PR, Brasil

**Keywords:** Limitación de la Movilidad, Ambulación Precoz, Modalidades de Fisioterapia, Pacientes Internos, Enfermería en Rehabilitación, Revisión

## Abstract

mapear la literatura científica referente al uso del *Perme Intensive Care Unit Mobility Score* en individuos adultos hospitalizados.

revisión de alcance, estructurada según las directrices metodológicas del *Joanna Briggs Institute - Evidence Synthesis Groups*, con búsquedas en siete bases de datos y literatura gris. Los estudios fueron seleccionados por dos revisores, utilizando un instrumento para la extracción de datos.

el análisis de los 29 estudios seleccionados mostró un predominio de estudios longitudinales (34,48%), realizados en Brasil (48,27%) en Unidades de Cuidados Intensivos (29%) y publicados entre 2020 y 2021 (48,24%). Los estudios evidenciaron el uso del Perme *Score* para la descripción y confiabilidad del instrumento, traducción y adaptación cultural, asociación entre la movilidad funcional, características clínicas y resultados, evaluación de la movilidad tras intervenciones, evaluación de la movilidad y potenciales barreras para la movilización, además del uso de la puntuación para la validación de otros instrumentos y perfiles clínicos diversos.

el Perme *Score* es un instrumento capaz de medir la movilidad física, incluyendo posibles barreras para la movilidad, con potencial para su uso en escenarios fuera de la Unidad de Cuidados Intensivos, en estudios de intervención para movilización temprana y predicción de resultados de la hospitalización.

## Destacados

**(1)** El Perme *Score* presenta un gran potencial de uso en diferentes escenarios. **(2)** Estudios posicionan al enfermero en el contexto de la evaluación de la movilidad funcional. **(3)** El Perme *Score* se ha empleado en protocolos de intervención para la movilización temprana.

## Introducción

La institución hospitalaria es considerada un espacio que proporciona alivio de síntomas, recuperación de la salud y acceso a diagnóstico. Sin embargo, dependiendo de los cuidados y tratamientos brindados, así como de otros diversos aspectos, la hospitalización puede convertirse en un factor desestabilizador, resultando en una mayor deterioración clínica y funcional, aumento del tiempo de estancia, descompensación de las multimorbilidades y un mayor riesgo de mortalidad^(1)^.

Las consecuencias del declive de la movilización durante la hospitalización pueden extenderse hasta cinco años después del alta, siendo resultado de hospitalizaciones prolongadas que se asocian con la edad, la gravedad de la enfermedad y el tipo de admisión (aguda/electiva). Estas fueron las conclusiones de un estudio de cohorte realizado con 10.430 individuos, en el grupo de Unidad de Cuidados Intensivos (UCI) (n= 5.215) y en la enfermería (n= 5.215), con una mediana de edad de 60 años (rango de 44 a 72) en Edimburgo (Reino Unido). Se observó una asociación del grupo UCI con una mayor mortalidad (RR 1,33; IC95%, 1,22-1,46, p= 0,001), mayor costo ($25.608 vs. $16.913/paciente^1^) y un mayor tiempo de hospitalización (51%)^(2)^.

Se estima un desacondicionamiento físico del 30% asociado a la hospitalización^(3)^. De este modo, el deterioro funcional no está relacionado únicamente con el cuadro clínico que motivó la hospitalización, así como la recuperación no es automática tras la resolución del problema que la provocó^(4)^. Cabe destacar que la movilidad funcional es un predictor de salud. Por lo tanto, la evaluación de las funciones locomotoras en el contexto hospitalario y la comprensión de las barreras para la movilización temprana se vuelven imprescindibles.

La movilización temprana debe entenderse como parte del proceso de rehabilitación del paciente hospitalizado, principalmente en la UCI, minimizando la debilidad muscular y el deterioro de la función física^(5)^. Existen diversas escalas que evalúan aspectos funcionales en la UCI, como el *Physical Function in Intensive care Test scored*, *Chelsea Critical Care Physical Assessment tool*, Perme *Intensive Care Unit Mobility Score*, *Surgical intensivecare unit Optimal Mobilization Score*, *ICU Mobility Scale* y *Functional Status Score for the ICU* ^(6)^. Ninguna de ellas ha sido considerada como el “estándar de oro” para la cuantificación de la movilidad funcional, ya que deberían ser de aplicación rápida y objetiva. Sin embargo, el Perme *Intensive Care Unit Mobility Score* tiene en cuenta las condiciones extrínsecas al paciente que interfieren en su movilidad en cama, tales como la presencia de accesos, tubos y drenajes torácicos, que pueden interpretarse como barreras para la movilización. La presencia de estos dispositivos no se puntúa ni se considera en la mayoría de las escalas.

El Perme *Intensive Care Unit Mobility Score*, concebido por Christiane Strambi Perme y aquí denominado Perme *Score*, fue traducido y adaptado al portugués. Está compuesto por siete categorías que abarcan el estado mental, las potenciales barreras para la movilidad, la fuerza funcional, la movilidad en cama, las transferencias, los dispositivos de ayuda para la deambulación y las medidas de resistencia. La puntuación varía de 0 a 32 puntos; una puntuación más alta indica mayor movilización y menor necesidad de asistencia, mientras que una puntuación baja indica menor movilización y mayor necesidad de asistencia^(7)^.

A pesar de ser un instrumento relativamente nuevo, ya traducido a diferentes idiomas y aportar como innovación la evaluación de las posibles barreras para la movilidad en la UCI, es indispensable representar las publicaciones sobre el puntaje en la literatura, considerando diferentes condiciones clínicas y escenarios. Tras una búsqueda preliminar en bases públicas de registro de protocolos de revisiones y bases de datos en línea, y ante la inexistencia de revisiones sistemáticas o registros de protocolos sobre esta escala, se consideró imperioso el desarrollo de esta revisión, cuyo objetivo fue mapear la literatura científica referente al uso del Perme *Intensive Care Unit Mobility Score* en individuos adultos hospitalizados.

## Método

### Tipo de estudio

Se trata de una revisión de alcance, un método sistemático que identifica y sintetiza el conocimiento por medio de la literatura existente o emergente, mapea la extensión y el alcance del tema, la naturaleza de la literatura y determina posibles brechas^(8)^. La revisión se desarrolló siguiendo las recomendaciones del *JBI – Evidence Synthesis Groups,* método recomendado para revisiones de alcance^(9)^ *,* además de las directrices establecidas por el *Preferred Reporting Items for Systematic Reviews and Meta-Analyses Extension for Scoping Reviews* (PRISMA-ScR), y fue registrada en la *International Platform of Registered Systematic Review and Meta-analysis Protocols* (INPLASY), bajo el número 2023100031 (https://inplasy.com/inplasy-2023-10-0031).

De este modo, la revisión de alcance se operacionalizó en cinco etapas: 1- establecimiento de la pregunta de investigación; 2- identificación de estudios relevantes; 3- selección e inclusión de estudios; 4- organización de los datos; y 5- recopilación, síntesis y presentación de los resultados^(10-11)^. Así, se formuló la pregunta y los elementos principales de búsqueda de esta revisión utilizando la estrategia PCC (*P – Population or Patients; C – Concept; C – Context*)^(12)^, siendo P (individuos adultos), C (Perme *Intensive Care Unit Mobility Score*) y C (hospitalario). En el Contexto, el entorno hospitalario se refiere a cualquier área de atención dentro de la unidad hospitalaria.

A partir de esto, se formuló la siguiente pregunta: ¿Cuáles son las evidencias disponibles en la literatura sobre el uso del Perme *Intensive Care Unit Mobility Score* en individuos adultos hospitalizados?

### Criterios de selección

Con respecto a la elegibilidad de los estudios, los criterios de inclusión fueron: (1) ser investigaciones primarias y secundarias, empíricas, cuantitativas y cualitativas, de cualquier diseño o metodología; (2) contener las variables de interés “Perme *Score*” y “hospitalización”; (3) estar publicados en inglés, español, portugués o francés; y (4) haber sido publicados a partir de 2014, año de publicación de la escala. Los criterios de exclusión de los estudios fueron: cartas al editor, resúmenes en actas de congresos, disertaciones, tesis, monografías y estudios de caso que no presentaran la variable de interés de la investigación.

### Búsqueda y recolección de datos

Inicialmente, la estrategia de búsqueda se construyó a partir de la identificación de los descriptores *Medical Subject Headings* (MeSH): *mobility limitation, early ambulation, physical therapy modalities* y *inpatients*, asociados al término libre “Perme”. Posteriormente, estos descriptores se tradujeron a los términos específicos de cada base consultada, como Descriptores en Ciencias de la Salud (DeCS) y *Embase Subject Headings* (Emtree).

No obstante, las bases de datos y los portales tendieron a cero cuando los descriptores se asociaron al operador booleano “AND”. Ante esto, se buscó la asistencia de un profesional bibliotecario para minimizar la posibilidad de errores. Así, la estrategia de búsqueda se redefinió para incluir únicamente los términos libres: “Perme” OR “Perme *scale*” OR “Perme *score*”.

En octubre de 2023, se realizaron búsquedas en las siguientes bases de datos: Biblioteca Virtual en Salud*,* EMBASE, PEDro, PubMed, SciELO, Scopus y *Web of Science,* y los títulos y resúmenes de los artículos fueron enviados a dos revisores para evaluar la elegibilidad de manera independiente. Para la literatura gris, se optó por realizar la búsqueda en el Catálogo de Tesis y Disertaciones de la *Coordenação de Aperfeiçoamento de Pessoal de Nível Superior* (CAPES). Entre los estudios de literatura gris que cumplieron con los criterios de inclusión y exclusión, todos ya contaban con artículos publicados en revistas científicas, los cuales fueron incluidos en la revisión.

Los resultados de las búsquedas textuales fueron exportados y transferidos al gestor de referencias gratuito Mendeley^U+00AE^, una herramienta que permite el acceso a múltiples investigadores y organiza las referencias en carpetas separadas. Se eliminaron los duplicados, manteniéndose solamente uno de los títulos. Posteriormente, se realizó la lectura de los títulos y resúmenes, con la exclusión de artículos que no cumplían con los criterios de inclusión o que presentaban alguno de los criterios de exclusión. Finalmente, los textos seleccionados fueron puestos a disposición de los revisores para una lectura completa, finalizando el proceso de inclusión.

Los investigadores responsables de la revisión son profesionales con experiencia en gerontología y miembros de un Grupo Multiprofesional de Investigación sobre Personas Mayores.

Cabe destacar que se utilizó el coeficiente de concordancia Kappa para describir la intensidad de acuerdo entre los revisores, la cual se basa en el número de respuestas concordantes, es decir, la frecuencia con la que los resultados coinciden entre los revisores^(13)^. En este estudio, la concordancia bruta entre los revisores fue del 96,81%, lo que corresponde a un coeficiente Kappa superior a 0,90. Además, un tercer revisor evaluó las divergencias en la selección de los estudios para tomar la decisión final sobre su inclusión o exclusión, con el fin de minimizar el riesgo de sesgo.

El número de artículos encontrados en cada base de datos y la suma de todas las bases se registraron en el diagrama de flujo PRISMA^(14)^, así como el proceso de selección y los motivos de exclusión.

### Extracción y análisis de datos

La extracción y el análisis descriptivo de los datos se llevaron a cabo mediante un protocolo elaborado por los propios autores, que abarca los criterios de elegibilidad previamente definidos, conforme lo recomendado ampliamente por el JBI. En este sentido, se incluyeron: nombre del autor y año de publicación, revista científica, país de origen, objetivos, resultados, diseño del estudio y tamaño de la muestra.

Debido al método elegido, no fue necesario realizar una evaluación formal de la calidad metodológica de los estudios incluidos. Sin embargo, la adhesión a los ítems de evaluación del *checklist* PRISMA *Extension for Scoping Reviews* (PRISMA-ScR) buscó garantizar el rigor metodológico del contenido^(15)^. Finalmente, se llevó a cabo un análisis temático del contenido para identificar los puntos convergentes en la literatura, delimitar los puntos fuertes del tema y las brechas existentes.

### Aspectos éticos

Dado que los estudios utilizados son de dominio público, no fue necesario someterlos al Comité de Ética en Investigación, según la Resolución CNS n° 510, de 2016.

## Resultados

La búsqueda en los portales y bases de datos resultó en 1.873 estudios. De estos, 837 fueron excluidos por ser duplicados, utilizando el gestor de referencias gratuito Mendeley^U+00AE^. Tras eliminar los duplicados, los 1.036 artículos seleccionados para la lectura de títulos y resúmenes fueron organizados en una hoja de cálculo de Excel^U+00AE^ para continuar con los análisis. Luego, se excluyeron 985 artículos tras la lectura de los títulos y 16 tras la lectura de los resúmenes, quedando 35 estudios para la lectura completa. De estos, se excluyeron seis, resultando en 29 estudios para la revisión. Para minimizar el posible riesgo de sesgo en la selección de los estudios, los revisores organizaron las referencias en el gestor de referencias gratuito Mendeley^U+00AE^. El refinamiento fue realizado por dos evaluadores independientes, buscando una concordancia del 100%. Un tercer revisor evaluó las posibles divergencias surgidas en la selección de los estudios para la toma de decisión final sobre su inclusión o exclusión. Dichos procedimientos están representados en la Figura 1, en el diagrama de flujo del método *Preferred Reporting Items for Systematic Reviews and Meta-Analysis* (PRISMA), que ilustra la selección de los artículos de esta revisión^(14)^.

En la Figura 2, se observa el diseño metodológico, el enfoque del estudio, los lugares de realización y los perfiles clínicos, que son la información principal encontrada.

**Figura 1 -:** Diagrama de flujo PRISMA-ScR de selección de los estudios. Curitiba, PR, Brasil, 2024

**Figura 2 -:** Representación esquemática de los hallazgos de estudios que involucran el Perme *Intensive Care Unit Mobility Score*. Curitiba, PR, Brasil, 2024

La Figura 3 muestra la categorización de los artículos por autor/año de publicación, revista científica, país de origen, objetivos, resultados, diseño del estudio y tamaño de la muestra.

Entre los estudios analizados, hubo un predominio de publicaciones en el año 2021 (n= 8; 27,58%), seguido de 2020 (n= 7; 24,14%), 2022 y 2023 (n= 3; 10,34% cada año), 2014, 2018 y 2019 (n= 2; 6,9% cada año) y, finalmente, 2016 y 2017 (n= 1; 3,45% cada año).

Respecto a las revistas científicas, se observaron 25 diferentes, con predominio de Colombia Médica, *NeuroRehabilitation*, *PloS One* y *Revista Brasileira de Terapia Intensiva* (n= 2; 6,89% cada una). Los países que se destacaron como sede de los estudios fueron: Brasil (n= 14; 48,27%), Estados Unidos de América (n= 6; 20,68%), Colombia (n= 3; 10,35%), India y Turquía (n= 2; 6,9% cada uno), Alemania y Taiwán (n= 1; 3,45% cada uno). Todos los artículos (n= 29) se publicaron en inglés.

En cuanto al diseño metodológico, predominaron los estudios longitudinales (n= 10; 34,48%). Los demás fueron estudios metodológicos (n= 8; 27,58%), estudios de cohorte retrospectivos (n= 4; 13,79%), estudios transversales (n= 3; 10,35%), estudios de intervención (n= 2; 6,9%), ensayo clínico randomizado y ensayo clínico no randomizado (n= 1; 3,45% cada uno). En los estudios se observó un total de 3.399 participantes, con tamaños que variaron entre 18 y 949 participantes.

**Figura 3 -:** Caracterización de los estudios que conformaron la revisión de alcance (n = 29). Curitiba, PR, Brasil, 2024

**Autor/ Año**	**Revista científica**	**País de origen**	**Objetivos**	**Resultados**	**Diseño del estudio**	**Tamaño de la muestra**
Perme, et al., 2014 ^( 16 )^	*Methodist DeBakey Cardiovascular Journal*	EE. UU.*	Describir una herramienta que evalúa la movilidad en la UCI ^†^ y su confiabilidad, además de abordar su aplicación clínica.	- Perme *Score* ^‡^ compuesto por 15 ítems agrupados en las siguientes categorías: estado mental, potenciales barreras para la movilidad, fuerza funcional, movilidad en cama, transferencias, marcha y resistencia;	Estudio metodológico	35
				- Concordancia Kappa entre evaluadores: 94,29% (68,57%-100%). Los valores Kappa fueron: ítem 2 (estado mental: el paciente es capaz de seguir 2 de 3 órdenes), sin concordancia (κ= 0); ítem 1 (estado de alerta en el contacto inicial), concordancia razonable (κ= 0,21-0,40); ítems 5, 10, 12 y 13, concordancia moderada (κ= 0,41-0,60); ítems 4, 7, 9 y 11, concordancia sustancial (κ= 0,61-0,80); e ítems 3, 6, 8, 14 y 15, alta concordancia (κ= 0,81-1,00).		
Nawa, et al., 2014 ^( 17 )^	*Journal of Critical Care*	EE. UU.*	Determinar la confiabilidad entre evaluadores del Perme *Score* ^‡^ en pacientes hospitalizados en la UCI ^†^ Cardiovascular.	Los 15 ítems del puntaje fueron analizados de forma individual. La confiabilidad interevaluadores fue de: 1,0 para los ítems 2, 3, 5, 6, 7, 8, 13 y 15; 0,82 para el ítem 1; 0,80 para el ítem 4; 0,60 para el ítem 9; 0,72 para los ítems 10 y 11; 0,78 para el ítem 12; y 0,90 para el ítem 14. El coeficiente de correlación intraclase fue de 0,98 (IC95% 0,97-0,99).	Estudio metodológico	20
Kawaguchi, et al., 2016 ^( 7 )^	*Jornal Brasileiro de Pneumologia*	Brasil	Realizar la traducción, adaptación y validación transcultural del Perme *Score* ^‡^ e IMS ^§^ al portugués de Brasil.	Los instrumentos mostraron una excelente concordancia interobservadores (κ> 0,90) y confiabilidad (α> 0,90) en todos los dominios. Se observó una fuerte correlación positiva entre ambos instrumentos (r= 0,941; *p* < 0,001).	Estudio metodológico	103
Yang, et al., 2017 ^( 21 )^	*American Journal of Respiratory and Critical Care Medicine*	EE. UU.*	Identificar el Perme *Score* ^‡^ de los pacientes al alta de la UCI ^†^ y el destino final tras el alta.	La mediana del Perme *Score* ^‡^ para los pacientes dados de alta a casa fue de 29; atención domiciliaria, 12; hospital de rehabilitación, 26; centro de cuidados especializados, 13; y fallecimiento, 7. Los puntajes más elevados del Perme *Score* ^‡^ se asociaron con el alta a casa ( *p* < 0,05).	Estudio longitudinal prospectivo	157
Nydahl, et al., 2018 ^( 18 )^	*European Journal of Physiotherapy*	Alemania	Traducir el Perme *Score* ^‡^ al idioma alemán e investigar la confiabilidad interevaluadores entre fisioterapeutas y enfermeros.	La confiabilidad interevaluadores (enfermeros y fisioterapeutas) fue casi perfecta: 0,96 (IC95%: 0,93-0,97). Dos ítems obtuvieron un índice de confiabilidad inferior a 0,8: capacidad para seguir órdenes (0,73, IC95%: 0,55-0,89) y dolor (0,46, IC95%: 0,09-0,68). El tiempo de completado de la escala fue de aproximadamente 30 segundos. Primer estudio que compara la aplicación entre diferentes profesionales.	Estudio metodológico	58
Wilches Luna, et al., 2018 ^( 19 )^	Colombia M *é* dica	Colombia	Realizar la traducción, adaptación cultural y evaluación de la fiabilidad interevaluadores de las versiones en español del Perme *Score* ^‡^ e IMS ^§^ en pacientes de UCI ^†^ .	La confiabilidad interevaluadores de la IMS ^§^ estuvo entre 0,97 y 1,0, y para el Perme *Score* ^‡^ entre 0,99 y 1,0 en los dos momentos de las mediciones: hasta 24 horas tras la admisión y al alta de la UCI ^†^ .	Estudio metodológico	150
Moecke & Biscaro, 2019 ^( 22 )^	*Fisioterapia Brasil*	Brasil	Analizar el estado funcional de pacientes hospitalizados en UCI ^†^ y su asociación con los resultados funcionales y clínicos, así como la fuerza muscular.	- Perme *Score* ^‡^ : 8,18±3,99 al despertar de la sedación y 13,68 ± 6 al alta de la UCI ^†^ ;	Estudio longitudinal prospectivo	40
				- El estado funcional y la fragilidad se asocian con resultados clínicos (alta/transferencia y fallecimiento) ( *p* = 0,022 y *p* = 0,019);		
				- La MRC ^||^ se asoció con la puntuación del Perme *Score* ^‡^ al despertar ( *p* < 0,001) y al alta de la UCI ^†^ ( *p* = 0,002);		
				- Puntuaciones más altas del Perme *Score* ^‡^ se asocian con MRC ^||^ > 48.		
Pereira, et al., 2019 ^( 36 )^	*Revista Brasileira de Terapia Intensiva*	Brasil	Evaluar el poder predictivo del Perme *Score* ^‡^ para complicaciones en el postoperatorio de trasplante hepático.	- Perme *Score* ^‡^ en la admisión (5,5) y alta (23,6) de la UCI ^†^ , en la enfermería 28,2 ± 5, y en el alta hospitalaria 31,7±0,7;	Estudio observacional prospectivo	30
				- Al comparar la evaluación inicial en la enfermería y el alta hospitalaria, hubo una mejora del estado funcional, Perme *Score* ^‡^ 28,2 ± 5 a 31,7 ± 0,7, *p* < 0,001;		
				- El tiempo de VM ^¶^ se asoció con Perme *Score* ^‡^ al alta de la UCI ^†^ (r = -0,374; *p* = 0,042). Cuanto mayor el tiempo de VM ^¶^ , menor el Perme *Score* ^‡^ al alta de la UCI ^†^ ;		
				- El número de tratamientos fisioterapéuticos se asoció inversamente con el Perme *Score* ^‡^ en la UH ^**^ (r = -0,578; *p* = 0,001).		
Ozcan, et al., 2020 ^( 31 )^	*Disability and Rehabilitation*	Turquía	Traducir y adaptar transculturalmente el instrumento FSS-ICU ^††^ al turco y evaluar sus propiedades psicométricas.	- La consistencia interna fue alta (Cronbach’s α=0,949). La confiabilidad inter e intraevaluadores fue excelente (α= 0,955-0,996);	Estudio metodológico	50
				- La puntuación FSS-ICU ^††^ mostró correlaciones de moderadas a altas con otras medidas funcionales, como: Perme *Score* ^‡^ (Spearman’s r= 0,92), actividades de vida diaria de Katz (r= 0,80) y fuerza de prensión manual (r= 0,76–0,77).		
Cavalli, et al., 2020 ^( 40 )^	*Middle East Journal of Rehabilitation and Health Studies*	Brasil	Analizar los desenlaces de alta o fallecimiento de pacientes hospitalizados en la UCI ^†^ considerando edad, sexo, gravedad, hospitalización, comorbilidades, tiempo de permanencia en la UCI ^†^ y en el hospital.	- La edad promedio fue de 54,91 ± 19,7 años, con el 62,5% de sexo masculino, predominando afecciones neurológicas (34%), pacientes en VM ^¶^ (63,9%), con una estancia en la UCI ^†^ de 5,76 días, y Perme *Score* ^‡^ inicial de 4,48 ± 7,17. La puntuación inicial del Perme *Score* ^‡^ en casos de fallecimiento fue menor (0,57 ± 1,98) en comparación con los casos de alta hospitalaria (7,27±8,16), *p* < 0,0001. Además, los valores de uso de fármacos vasoactivos y sedantes fueron mayores en casos de fallecimiento, *p* ≤ 0,0001;	Estudio transversal	288
				- Hubo una diferencia significativa en la puntuación inicial del Perme *Score* ^‡^ según el tipo de hospitalización: pacientes clínicos 4,15 ± 7,30 y quirúrgicos 5,44±6,75, *p* < 0,01.		
Ceron, et al., 2020 ^( 38^ )	*Respiratory Care*	Brasil	Evaluar las alteraciones en el desempeño de la movilidad con el uso de válvulas fonadoras en individuos traqueostomizados.	- El Perme *Score* ^‡^ aumentó de 11,3 (IQR ^‡‡^ 10,1-12,0) a 18,2 (IQR ^‡‡^ 16,2-20,1) inmediatamente después del inicio del uso de la válvula fonadora, *p* < 0,01.	Estudio de cohorte prospectivo	18
				- Al evaluar las categorías del Perme *Score* ^‡^ , se observaron cambios en las puntuaciones de la “categoría de transferencia” (“sentarse para ponerse de pie”, “equilibrio estático en posición de pie una vez establecida” y “transferencia de la cama a la silla o de la silla a la cama”);		
				- El uso de válvulas fonadoras en traqueostomizados mejoró la movilidad.		
Gatty, et al., 2020 ^( 26 )^	*Physiotherapy Theory and Practice*	India	Estudiar la eficacia de un protocolo de movilización temprana en el estado de movilidad de pacientes en UCI ^†^ .	- Perme *Score* ^‡^ en el primer día de UCI ^†^ , mediana (IQR ^‡‡^ ): grupo intervención 6 (2,2-8) y grupo control 6 (3-7); en el primer día de rehabilitación, (IQR ^‡‡^ ): grupo intervención 7 (5-9) y grupo control 5 (3-8); y en el último día de rehabilitación, (IQR ^‡‡^ ): grupo intervención 18 (11-24,7) y grupo control 7 (5-11);	Ensayo clínico no randomizado	63
				- La diferencia en la puntuación del Perme *Score* ^‡^ entre el primer y el último día de rehabilitación fue de 9 (3,2-17) en el grupo intervención y 2 (0-5) en el grupo control, *p* ≤ 0,001;		
				- Hubo un aumento significativo de la mediana del Perme *Score* ^‡^ desde el primer día de internación en UCI ^†^ hasta el último día de rehabilitación, siendo 12,5 (6,2-7,7) en el grupo intervención y 2 (0-6) en el grupo control, *p* < 0,001;		
				- El Perme *Score* ^‡^ se comparó entre el primer día de UCI ^†^ y el primer día de rehabilitación, sin observarse diferencias en el grupo intervención ( *p* = 0,069) ni en el grupo control ( *p* = 0,124).		
Lima, et al., 2020 ^( 23 )^	*Fisioterapia em Movimento*	Brasil	Determinar la relación entre la movilidad funcional y los desenlaces clínicos de pacientes hospitalizados en UCI ^†^ .	- Los resultados clínicos (fallecimiento/alta) se asociaron con los dominios del Perme *Score* ^‡^ : estado mental ( *p* = 0,040), barreras para la movilidad ( *p* = 0,016), fuerza ( *p* = 0,01) y movilidad en cama ( *p* = 0,024). La puntuación total del Perme *Score* ^‡^ fue 0 (0-10) en el desenlace de fallecimiento y 10 (0-23) para el alta, ( *p* = 0,002);	Estudio longitudinal prospectivo	33
				- El uso de la VM ^¶^ estuvo asociado a puntuaciones bajas en la escala, 2,40±4,19, mientras que la ausencia de su uso se relacionó con puntuaciones más altas, 12,85±6,61, *p* = 0,000;		
				- Hubo una correlación positiva (R= 0,745) entre Perme *Score* ^‡^ y el nivel de sedación, y se encontró una correlación inversa entre APACHE ^§§^ y el Perme *Score* ^‡^ (R= -0,526), y entre APACHE ^§§^ y días de VM ^¶^ (R= -0,602).		
Perme, et al., 2020 ^( 41 )^	*Journal of Acute Care Physical Therapy*	EE. UU.*	Evaluar la asociación entre los instrumentos Perme *Score* ^‡^ , MRC-SS ^||||^ y el desenlace clínico de pacientes internados en UCI ^†^ .	- La media del Perme *Score* ^†^ fue de 23,56 (±7,09);	Estudio longitudinal prospectivo	250
				- El Perme *Score* ^†^ según el destino del alta fue: domicilio 26,05±5,42, instituciones de cuidados prolongados 18,65±8,43, servicios de enfermería especializados 17,38±7,72, rehabilitación 20,3±7,48, y otros 18±5,8;		
				- El Perme *Score* ^‡^ de los pacientes dados de alta fue diferente de aquellos derivados a rehabilitación ( *p* < 0,001), servicio de enfermaría especializado ( *p* < 0,001), cuidados agudos a largo plazo ( *p* < 0,001), y cuidados paliativos/hospicio o fallecidos ( *p* < 0,001);		
				- Existe una correlación moderada entre MRC-SS ^||||^ y Perme *Score* ^‡^ (r= 0,66; *p* < 0,001);		
				- Los pacientes en UCI ^†^ con una mayor puntuación en el Perme *Score* ^‡^ o MRC-SS ^||||^ al momento de la evaluación del fisioterapeuta fueron dados de alta a casa, mientras que aquellos con puntuaciones más bajas requirieron cuidados postagudos;		
				- Un Perme *Score* ^‡^ o MRC-SS ^||||^ más elevado indicó una mayor probabilidad de alta a casa.		
Pinto, et al., 2020 ^( 29 )^	*Critical Reviews™ in Physical and Rehabilitation Medicine*	India	Identificar PBM ^¶¶^ y evaluar el cambio en la movilidad durante la estancia en la UCI ^†^ en pacientes intoxicados por organofosforados.	- El Perme *Score* ^‡^ (2,50–6,0; *p* ≤0,01) mejoró en los subcomponentes movilidad en cama (1,83±0,35 a 7,23±3,84), transferencias (0,37±0,02 a 4,78±3,84) y marcha (1,83±0,35 a 5,23±1,27) del día 2 al día 10 de hospitalización;	Estudio transversal	37
				- El tubo endotraqueal y la infusión continua de medicamentos fueron identificados como las PBM ^¶¶^ (66,6%) según el Perme *Score* ^‡^ .		
Wilches Luna, et al., 2021 ^( 42 )^	*Physiotherapy Research International*	Colombia	Determinar el CMD ^***^ y la capacidad de respuesta del Perme *Score* ^‡^ en adultos internados en UCI ^†^ .	- El valor del CMD ^***^ para el Perme *Score* ^‡^ fue de 1,36, lo que demuestra que los adultos internados en la UCI ^†^ que presentaron una diferencia superior a 1,36 puntos entre la primera y la segunda evaluación mostraron una diferencia mínima detectable;	Estudio longitudinal prospectivo	142
				- El CMD ^***^ se observó en el 80% de los pacientes;		
				- Hubo una diferencia significativa en el tiempo de VM ^¶^ (0,011) y en el tiempo de estancia en la UCI ^†^ ( *p* < 0,04).		
Cordeiro, et al., 2021 ^( 37 )^	*Journal of Clinical and Translational Research*	Brasil	Evaluar la asociación entre deambulación precoz y funcionalidad en pacientes sometidos a cirugía de sustitución valvular cardíaca.	- El grupo que caminaba presentó una disminución de 11±2 en el Perme *Score* ^‡^ , mientras que el grupo que no caminaba mostró una disminución de 13±2, *p* =0,34.	Estudio de cohorte prospectivo	170
Da Rosa, et al., 2021 ^( 27 )^	*NeuroRehabilitation*	Brasil	Verificar la asociación entre el entrenamiento aeróbico en ciclismo y la fuerza muscular de los miembros inferiores, la velocidad de marcha, el equilibrio, la movilidad y la funcionalidad de individuos con ACV ^†††^ .	- Intervención: entrenamiento aeróbico en ciclismo;	Ensayo clínico randomizado	20
				- En el análisis de la fuerza muscular de los miembros inferiores, hubo una mejora intergrupal entre pre y post-intervención. Se observó una mejora significativa en todos los grupos musculares, incluido el lado parético y no parético, solamente en el GI ^‡‡‡^ ;		
				- En el TC10m ^§§§^ y en la EEB ^||||||^ hubo una diferencia intragrupal en el GI ^‡‡‡^ , *p* <0,001, y una diferencia intergrupal con mejor resultado para el GI ^‡‡‡^ , *p* < 0,001. Se observó que el GI ^‡‡‡^ mejoró en equilibrio (pre 0 ± 0, post 28,9±7,45) y en velocidad de marcha (pre 0±0, post 0,67±0,78);		
				- Con relación al Perme *Score* ^‡^ , hubo una diferencia entre los resultados del GI ^‡‡‡^ (pre 14,3 ±3,10 y post 27,3±2,91, *p* < 0,001) y del GC ^****^ (pre 9,40±2,31 y post 12,30±3,40, *p* < 0,001).		
				- Hubo mayor movilidad en individuos después de un ACV ^†††^ agudo sometidos al cicloergómetro.		
Luna, Perme e Gastaldi, 2021 ^( 30 )^	*Canadian Journal of Respiratory Therapy*	Colombia	Identificar las PBM ^¶¶^ tempranas en adultos mediante el Perme *Score* ^‡^ y su asociación con los días en VM ^¶^ y el tiempo de estancia en la UCI ^†^ .	- Se observaron correlaciones inversas entre el total de días en VM ^¶^ y la puntuación total de PBM ^¶¶^ al ingreso (r= -0,773; *p* < 0,05) y al alta (r = -0,559; *p* < 0,05) de la UCI ^†^ , así como entre el tiempo de estancia en la UCI ^†^ y la puntuación total de PBM ^¶¶^ al ingreso (r = -0,420; *p* < 0,05) y al alta (r= 0,283; *p* < 0,05) de la UCI ^†^ ;	Estudio observacional prospectivo	142
				- Hubo correlación entre la puntuación total del ítem barreras y la puntuación total del Perme *Score* ^‡^ (r= -0,91; *p* < 0,01).		
Özsoy, et al., 2021 ^( 32 )^	*Turkish Journal of Medical Sciences*	Turquía	Realizar la traducción y adaptación cultural de la IMS ^§^ al turco y analizar sus propiedades psicométricas.	- La confiabilidad inter e intraevaluadores de la IMS ^§^ fue excelente, con un índice de Kappa de 0,87 (0,80-0,93) y 0,92 (0,87-0,96);	Estudio metodológico	70
				- Se encontraron correlaciones significativas de la escala IMS ^§^ con la FSS-ICU ^††^ , Perme *Score* ^‡^ , Katz, FPM ^††††^ derecha e izquierda (rs≥ 0,60; *p* < 0,05);		
				- Ambiente de UCI ^†^ , edad media de 69,65±10,73, 60% del sexo masculino, diagnóstico principal al ingreso síndrome coronario agudo 54,3%, Perme *Score* ^‡^ 21,32±5,09.		
Souza, et al., 2021 ^( 28 )^	*Revista Pesquisa em Fisioterapia*	Brasil	Evaluar la oferta energética y proteica en pacientes críticos sometidos a fisioterapia convencional combinada con cicloergómetro activo (GI ^‡‡‡^ ) o fisioterapia convencional (GC ^****^ ) y correlacionarla con MRC ^||^ , datos antropométricos y Perme *Score* ^‡^ .	- El nivel de adecuación calórica y proteica en ambos grupos fue del 73,9% y 69,5%, respectivamente. En el GC ^****^ , la adecuación calórica fue del 66,6% y la proteica del 58,3%, mientras que en el GI ^‡‡‡^ la adecuación fue del 81,8% tanto calórica como proteica;	Estudio de intervención	23
				- Hubo una reducción significativa de la circunferencia de la pantorrilla al 9º día en comparación con la inicial, *p* = 0,001. No se observó diferencia en el GC ^****^ ni en el GI ^‡‡‡^ , p= 0,053;		
				- Hubo una reducción significativa de la circunferencia del brazo al 9º día en comparación con la inicial en ambos grupos (GC ^****^ *p* = 0,038 y GI ^‡‡‡^ *p* = 0,041);		
				- Se encontró una correlación moderada e inversa entre el déficit energético y el Perme *Score* ^‡^ final en el GC ^****^ (r= 0,59; *p* = 0,03).		
Timenetsky, et al., 2021 ^( 34 )^	*PLoS One*	Brasil	Describir el nivel de movilidad de pacientes con COVID-19 ingresados en la UCI ^†^ y analizar los factores asociados al nivel de movilidad al momento del alta de la UCI ^†^ .	- El Perme *Score* ^‡^ mejoró al comparar el alta 20,0 (7-28) con el ingreso 7,0 (0-16) en la UCI ^†^ , *p* < 0,001;	Estudio de cohorte retrospectivo	136
				- Hubo una mejora de la movilidad durante la estancia en la UCI ^†^ en el 64,7% de los pacientes, y la mediana del Perme *Score* ^‡^ fue de 1,5 (0,6-3,4);		
				- El grupo con mejoría tuvo una menor duración de VM ^¶^ : 10 (5-14) vs. 15 (8-24) días, *p* = 0,021; menor tiempo de internación: 25 (12-37) vs. 30 (11-48) días, p< 0,001; y menor mortalidad en UCI ^†^ y en el hospital;		
				- Los predictores independientes para la movilidad fueron menor edad, menor Índice de Comorbilidad de Charlson y no haber recibido terapia de reemplazo renal.		
Reis, et al., 2021 ^( 33 )^	*Revista Brasileira de Terapia Intensiva*	Brasil	Traducir y adaptar transculturalmente al portugués de Brasil el *Early Rehabilitation Index* y verificar las propiedades psicométricas de la herramienta (ERBI ^‡‡‡‡^ ) al alta de la UCI ^†^ .	- El ERBI ^‡‡‡‡^ mostró una confiabilidad adecuada, con un coeficiente alfa de Cronbach de 0,65;	Estudio metodológico	122
				- La confiabilidad entre evaluadores fue excelente, con un coeficiente de 0,94 (IC95% 0,92-0,96);		
				- Se observó la validez del ERBI ^‡‡‡‡^ mediante correlaciones fuertes y significativas con la puntuación total del Perme *Score* ^‡^ (rô= 0,72);		
				- Características de la UCI ^†^ : Perme *Score* ^‡^ 25,5 (15-30), edad promedio de 56 años (46,8-66), sexo masculino 51%, motivo de ingreso (23% sepsis, 19% postoperatorio electivo, 14% trastorno cardiovascular), 68% VM ^¶^ , tiempo de VM ^¶^ de 5 (3-8) días, y alta hospitalaria del 88%.		
Nawa, et al., 2022 ^( 20 )^	Colombia M *é* dica	Brasil	Evaluar la influencia del Perme *Score* ^‡^ en el tiempo de permanencia en la UCI ^†^ en el postoperatorio de cirugía cardíaca e investigar la asociación de las variables preoperatorias con la movilidad postquirúrgica.	- El Perme *Score* ^‡^ en los días 2 y 3 estuvo asociado con el tiempo de permanencia en la UCI ^†^ : (β= -0,76; IC95% -1,19 a -0,33, *p* = 0,001) y (β= -2,67; IC95% -3,38 a -1,95, *p* < 0,001), respectivamente.	Estudio longitudinal prospectivo	44
				- Un aumento de 4,6 puntos en el Perme *Score* ^‡^ redujo un día el tiempo de estancia en la UCI ^†^ , independientemente del procedimiento quirúrgico;		
				- La función pulmonar preoperatoria fue uno de los principales predictores independientes del estado de movilidad en los primeros tres días de internación en la UCI ^†^ , así como la fracción de eyección del ventrículo izquierdo y el tiempo de circulación extracorpórea en el día 1 ( *p* = 0,006), la edad y la fracción de eyección del ventrículo izquierdo en el día 2 ( *p* = 0,002), y la Presión Espiratoria Máxima en el día 3 ( *p* < 0,001).		
Nawa, et al., 2022 ^( 35 )^	*PLoS One*	EE. UU.*	Abordar variaciones en las características clínicas, uso de VM ^¶^ , y factores de riesgo asociados con el nivel de movilidad durante la estancia en la UCI ^†^ de pacientes gravemente enfermos con COVID-19.	- La tasa general de pacientes que lograron salir de la cama fue del 63,3%, y el 20,5% fueron capaces de caminar 30 metros al alta de la UCI ^†^ ;	Estudio de cohorte retrospectivo	949
				- El porcentaje de pacientes que lograron salir de la cama durante la estancia en la UCI ^†^ fue menor entre aquellos en VM ^¶|^ (36,4% vs. 72%, *p* < 0,001), los pacientes de mayor edad (51,2% vs. 64,1%, *p* < 0,001) y los pacientes frágiles (41,2% vs. 56,5% en pre-frágiles vs. 65,7% en no frágiles; *p* < 0,001);		
				- Tras el ajuste por factores de confusión, los predictores independientes de mejora en el nivel de movilidad fueron la fragilidad (OR 0,52; IC95%: 0,29-0,94; *p* = 0,03) y un mayor Perme *Score* ^‡^ al ingreso (OR 0,35; IC95% 0,28-0,43, *p* < 0,001).		
Yen, et al., 2022 ^( 39 )^	*NeuroRehabilitation*	Taiwán	Investigar los efectos de la movilización progresiva temprana en la movilidad funcional y en la tasa de movilidad fuera de la cama alcanzada por pacientes con traumatismo craneoencefálico moderado a grave.	- Al alta de la UCI ^†^ , los pacientes del GI ^‡‡‡^ (movilización progresiva temprana) se encontraban en el Nivel 1 (9,5%), Nivel 2 (33,3%) y Nivel 3 (52,4%), mientras que los pacientes del GC ^****^ permanecieron en el Nivel 0 en la Escala Modificada de Movilidad en la UCI ^†^ ;	Estudio de intervención	86
				- Perme *Score* ^‡^ inicial en el GC ^****^ 3,23±2,03 vs. 2,83±2,26 en el GI ^‡‡‡^ ;		
				- El GI ^‡‡‡^ presentó un aumento significativo en el Perme *Score* ^‡^ total al momento del alta de la UCI ^†^ (GI ^‡‡‡^ 6,62±4,33 vs. GC ^****^ 3,64±1,66, *p* = 0,001, η2p= 0,995);		
				- Al alta de la UCI ^†^ , solamente el subpuntaje del nivel de asistencia necesaria para la movilidad del Perme *Score* ^‡^ mostró diferencia (GC ^****^ 3,64±1,66 vs. GI ^‡‡‡^ 6,62±4,33, *p* = 0,001).		
Nascimento, et al., 2023 ^( 24 )^	*Canadian Journal of Respiratory Therapy*	Brasil	Evaluar la movilidad de pacientes con COVID-19 mediante el Perme *Score* ^‡^ fuera del entorno de la UCI ^†^ y correlacionar el valor del puntaje con el tiempo de hospitalización.	- Aumento promedio de 7,3 puntos (IC95% 5,7-8,8, *p* < 0,001) entre los momentos de admisión en la UH ^**^ y el alta hospitalaria;	Estudio de cohorte retrospectivo	69
				- Los valores promedio del Perme *Score* ^‡^ en la admisión a la UH ^**^ fue de 17,5 (15,8-19,3) y en el alta hospitalaria de 24,8 (23,3-26,3);		
				- No hubo asociación entre los valores del Perme *Score* ^‡^ y el tiempo de hospitalización (0,929 IC95%; 0,861-1,002, *p* = 0,058);		
				- Se observó que el 17,9% de los pacientes alcanzaron la puntuación máxima en el Perme *Score* ^‡^ , de los cuales solamente uno (1,4%) obtuvo la puntuación máxima tanto al ingreso como al alta.		
Rittel, et al., 2023 ^( 43 )^	*Dimensions of Critical Care Nursing*	EE. UU.*	Evaluar la movilidad y el autocuidado entre personas mayores hospitalizadas en UCI ^†^ e identificar barreras para la intervención temprana.	- Perme *Score* ^‡^ inicial (IQR ^‡‡^ 25-75) 23 (11,5-28) y final (IQR ^‡‡^ 25-75) 27 (16-31);	Estudio de cohorte retrospectivo	43
				- Del total de pacientes, el 76% presentó una mejora en el Perme *Score* ^‡^ ;		
				- De los pacientes con mejora en el Perme *Score* ^‡^ , la mediana fue (IQR ^‡‡^ 25-75) 9,4 (3,1-15,6);		
				- Las razones para no movilización fueron: falta de personal o tiempo suficiente 17%, incapacidad mental para seguir instrucciones 8%, estar bajo sedación activa 6%, inestabilidad hemodinámica 3% y transición a cuidados de confort/hospicio 3%.		
Tavares, et al., 2023 ^( 25 )^	*Heart & Lung - The Journal of Cardiopulmonary and Acute Care*	Brasil	Evaluar la fuerza, la movilidad y la debilidad adquirida en UCI ^†^ entre individuos con y sin COVID-19, y determinar el punto de corte del Perme *Score* ^‡^ para ICUAW ^§§§§^ .	- Perme *Score* ^‡^ al alta de la UCI ^†^ , Grupo COVID-19 18,1 (15,5-20,7) vs. Grupo no COVID-19 18,3 (15-21,5), *p* = 0,20;	Estudio transversal	48
				- Perme *Score* ^‡^ al alta hospitalaria Grupo COVID-19 27,2 (24,6-29,9) vs. Grupo no COVID-19 27,6 (24,4-30.8), *p* = 0,65;		
				- El aumento de una unidad en el Perme *Score* ^‡^ redujo el tiempo de internación en 1,04 días para otras patologías y en 8,30 días para COVID-19;		
				- El punto de corte con mayor sensibilidad (0,82) y especificidad (0,70) para la detección de ICUAW ^§§§§^ en el Perme *Score* ^‡^ fue de 18 puntos.		

*EE.UU. = Estados Unidos de América; ^†^UCI = Unidad de Cuidados Intensivos; ^‡^Perme *Score* = Perme *Intensive Care Unit Mobility Score*; ^§^IMS = *ICU Mobility Scale*; ^||^MRC *= Medical Research Council*; ^¶^VM = Ventilação Mecânica; **UH = Unidad de Hospitalización; ^††^FSS-ICU = *Functional Status Score for the Intensive Care Unit*; ^‡‡^IQR = *Interquartile range*; ^§§^APACHE = *Acute Physiology and Chronic Health disease Classification System*; ^||||^MRC-SS = *Medical Research Council Sum Score*; ^¶¶^PBM = Potenciales Barreras para la Movilización; ***CMD = Cambio Mínimo Detectable; ^†††^ACV = Accidente Cerebrovascular; ^‡‡‡^GI = Grupo Intervención; ^§§§^TC10m = Test de Caminata de 10 metros; ^||||||^EEB = Escala de Equilibrio de Berg; ****GC = Grupo Control; ^††††^FPM = Fuerza de Prensión Manual; ^‡‡‡‡^ERBI = *Early Rehabilitation Barthel Index*; ^§§§§^ICUAW = Debilidad Adquirida en la UCI

Los estudios abordaron una variedad de temas, como la descripción y confiabilidad del instrumento diseñado por Christiane Strambi Perme^(16-17)^; traducción y adaptación cultural para otros idiomas^(5,18-19)^; asociación entre movilidad funcional y características clínicas^(20)^; evaluación de la movilidad y el desenlace de los pacientes^(21-25,30)^; evaluación de la movilidad tras intervenciones específicas, como el protocolo de movilización temprana^(26)^, comparación entre fisioterapia convencional y cicloergómetro^(27-28)^; evaluación de la movilidad y potenciales barreras para la movilización^(29-30)^; y uso del Perme *Score* como herramienta de validación para otros instrumentos^(31-33)^.

La mayoría de los estudios tuvo como escenario principal la UCI. Solamente dos estudios se desarrollaron en unidades de hospitalización (enfermería)^(24,36)^. Otra información relevante se refiere a los perfiles específicos de los pacientes evaluados, que incluyeron COVID-19^(24,34-35)^, trasplante hepático^(36)^, cirugía cardíaca^(37)^, traqueostomizados^(38)^ e intoxicados por organofosforados^(29)^.

## Discusión

El instrumento Perme *Intensive Care Unit Mobility Score* fue desarrollado para la evaluación de la movilidad funcional y está compuesto por subcategorías: estado mental, potenciales barreras para la movilidad, fuerza funcional, movilidad en cama, transferencias, marcha y resistencia. El instrumento mostró una concordancia del 94,29% (68,57%-100%) entre los evaluadores^(16)^, con su confiabilidad evaluada en una UCI cardiovascular ese mismo año^(17)^.

Tras la evaluación de la confiabilidad del instrumento, este fue traducido y validado al portugués de Brasil^(5)^, al español^(19)^ y al alemán^(18)^, que además de la traducción y validación transcultural, se obtuvo una confiabilidad entre evaluadores (fisioterapeutas y enfermeros) del 96% (93-97%), destacando el papel del profesional de enfermería en la evaluación de la movilidad funcional^(18)^.

Con relación a las publicaciones sobre el Perme *Score*, los primeros estudios describieron la puntuación del Perme *Score*. Posteriormente, surgieron investigaciones enfocadas en condiciones clínicas específicas, complicaciones postoperatorias y resultados clínicos. Se observó que valores más elevados en el Perme *Score* estaban asociados con el alta hospitalaria al domicilio^(21)^.

Diferentes valores del Perme *Score* orientan las derivaciones al alta hospitalaria. Los pacientes dados de alta a domicilio presentaron un Perme *Score* de 29; para atención domiciliaria, 12; para hospital de rehabilitación, 26; para servicios de enfermería especializados, 13; y para fallecimiento, 7^(21)^. Se observó un perfil muy similar de destinos en pacientes internados en UCI en los Estados Unidos de América: domicilio 26,05 (±5,42), instituciones de cuidado prolongado 18,65 (±8,43), servicios de enfermería especializados 17,38 (±7,72), y rehabilitación 20,3±7,48(41). En vista de ello, el Perme *Score* demuestra ser una herramienta útil para evaluar la efectividad de la rehabilitación intrahospitalaria, orientando los esfuerzos hacia una derivación adecuada del paciente en la Red de Atención de Salud (RAS), con el fin de mejorar la movilidad y, en consecuencia, alcanzar mejores resultados.

La puntuación del Perme *Score* en el desenlace de fallecimiento fue menor (0,57; ±1,98) en comparación con la puntuación al alta hospitalaria (7,27; ±8,16), *p*< 0,0001, y los valores de uso de drogas vasoactivas y sedantes fueron superiores en el grupo de fallecimiento, *p*≤ 0,0001. Además, las hospitalizaciones clínicas presentaron un Perme *Score* inferior a las hospitalizaciones quirúrgicas (4,15 ± 7,30 vs. 5,44±6,75, *p*< 0,01)^(40)^. Considerando que la inmovilidad está asociada a una serie de desenlaces negativos, la evaluación de la movilidad, así como la implementación de un programa de movilización temprana, deben ser instauradas en la gestión del cuidado.

Aunque la mayoría de los estudios aplicaron el Perme *Score* en la UCI, algunos lo utilizaron en Unidades de Hospitalización (UH). Se observó un aumento promedio de la movilidad funcional en las UH (7,3; IC95% 5,7-8,8, *p*< 0,001) entre los momentos de admisión y alta hospitalaria. Los valores promedio del Perme *Score* en la admisión en la UH fueron de 17,5 (IC95% 15,8-19,3), y al alta hospitalaria de 24,8 (IC95% 23,3-26,3)^(24)^. Estos estudios amplían el uso del Perme *Score* más allá de la UCI, demostrando la viabilidad de aplicar esta herramienta en la gestión de la movilidad en diferentes escenarios de atención en salud.

El uso de protocolos de movilización temprana en el estado de movilidad funcional de los pacientes en la UCI contribuye a un aumento significativo del Perme *Score* desde el primer día de internación hasta el último día de rehabilitación^(26)^. La intervención de deambulación precoz también fue objeto de estudio, en el cual los pacientes que deambularon de forma temprana presentaron una menor disminución del Perme *Score* tras cirugía de válvula cardíaca^(37)^. Asimismo, la movilización temprana demostró mejores resultados en la movilidad funcional de pacientes con traumatismo craneoencefálico (TCE) moderado a grave. El Grupo Intervención (GI) presentó un aumento significativo en el Perme *Score* al alta de la UCI en comparación con el Grupo Control (GC) (GI 6,62 ±4,33 vs. GC 3,64 ±1,66, *p*= 0,001)^(39)^.

En este mismo sentido, el uso del cicloergómetro en la rehabilitación tras un accidente cerebrovascular agudo demostró una mayor movilidad y funcionalidad en comparación con la fisioterapia convencional^(27)^. La movilización temprana debe tener como principio la reinserción social, con el objetivo de minimizar o revertir los impactos de la hospitalización mediante la realización de actividades que promuevan la independencia. Por ello, la movilización temprana debe ser una meta para todo el equipo multidisciplinario.

Las potenciales barreras para la movilización deben ser evaluadas por el equipo multidisciplinario y, una vez identificadas, es posible implementar estrategias para minimizarlas. Puntuaciones más altas en las Potenciales Barreras para la Movilización se asociaron con mayores valores del Perme *Score*, menor tiempo de permanencia en ventilación mecánica y menor tiempo total de estancia en la UCI^(30)^.

Un estudio realizado en los EE. UU. tuvo como objetivo evaluar la movilidad y el autocuidado de adultos mayores en la UCI, además de identificar barreras para la movilización temprana. Se observó un Perme *Score* inicial de 23 (IQR 11,5-28) y final de 27 (IQR 16-31), con un 76% de los pacientes mostrando mejoría. Las razones para no realizar una movilización temprana incluyeron la falta de personal o de tiempo suficiente (17%), incapacidad cognitiva para seguir instrucciones (8%), sedación (6%), inestabilidad hemodinámica (3%) y transición a cuidados de confort/hospicio (3%)^(43)^.

El Perme *Score* mostró poder predictivo en el postoperatorio de trasplante hepático, con una asociación inversa entre la puntuación y el tiempo de ventilación mecánica (*p*= 0,042), así como con el número de intervenciones fisioterapéuticas en la unidad de hospitalización (*p*= 0,001)^(36)^.

El desempeño de la movilidad funcional se evidenció tras el uso de la válvula fonadora en individuos traqueostomizados, con un aumento del Perme *Score* de 11,3 (IQR 10,1-12,0) a 18,2 (IQR 16,2-20,1); *p*< 0,01. Se discute ampliamente sobre los beneficios de la válvula fonadora en el proceso del habla y en la regulación de patrones respiratorios más cercanos a lo fisiológico, así como en el proceso de deglución. Sin embargo, se aborda poco sobre los beneficios indirectos de la válvula fonadora, que, cuando se introduce poco después de la suspensión de la ventilación mecánica en individuos traqueostomizados, puede mejorar la capacidad de movilidad^(38)^.

El Perme *Score* se empleó como comparativo en la traducción y adaptación transcultural del *Functional Status Score for the Intensive Care Unit* (FSS-ICU)^(31)^ y de la *ICU mobility scale (IMS)* al turco^(32)^. La validación del Perme *Score* al turco no se incluyó en esta revisión, ya que dicho trabajo no fue publicado, estando disponible únicamente en la base de disertaciones de la *Muğla Sıtkı Koçman University, en idioma turco.* Además de estos estudios, el Perme *Score* también se utilizó en la traducción del *Early Rehabilitation Index* (ERI) al portugués de Brasil^(33)^. Por ser un instrumento de fácil aplicación, que considera las potenciales barreras para la movilidad, ha sido empleado como índice de comparación para nuevos instrumentos.

Es importante señalar que no se encontraron en la literatura pruebas adicionales para la validación de las propiedades psicométricas ni la validez del constructo. Aunque no es obligatorio, es altamente recomendable que, tras el proceso de traducción y adaptación, los investigadores aseguren que la nueva versión demuestre las propiedades de medición necesarias para su aplicación prevista. De este modo, se tendría mayor confianza en que el instrumento adaptado mide un constructo comparable al original^(44)^.

Como limitación del estudio, se destaca la ausencia de validación psicométrica de la escala. Además, el método elegido busca mapear la literatura en un determinado campo de interés, sin tener como objetivo encontrar la mejor evidencia de una intervención en salud. Por lo tanto, no fue posible clasificar la solidez de la evidencia, solamente rastrearla y anticipar sus potencialidades. A pesar de estas limitaciones, este estudio tiene como punto destacable la estrategia de búsqueda amplia y el proceso estandarizado de extracción de datos requerido por el JBI. Se sugiere la realización de otros estudios con el fin de llevar a cabo pruebas adicionales de la versión adaptada al portugués de Brasil, permitiendo el uso de la escala en otros entornos, además de la UCI, como ya se ha demostrado.

Como aporte al conocimiento científico en el área de la salud, se destaca la posibilidad de utilizar el Perme *Score* en otros sectores hospitalarios, ayudando a medir la movilidad funcional de forma rápida y objetiva, incluso considerando condiciones extrínsecas al paciente.

## Conclusión

Esta revisión de alcance mapeó la aplicación del Perme *Score* en la descripción y evaluación de la confiabilidad del instrumento, así como su uso como estándar en la traducción y adaptación cultural de otros instrumentos. Asimismo, abordó la evaluación de la movilidad funcional asociada a características clínicas, resultados clínicos y potenciales barreras para la movilización. También se destacó el uso del Perme *Score* en protocolos de intervención en movilización temprana, en la evaluación de la movilidad funcional en fisioterapia convencional y en el uso del cicloergómetro.

Las diferentes puntuaciones en la escala de movilidad funcional se asociaron con características clínicas y sus desenlaces, intervenciones y potenciales barreras para la movilidad.

Aunque el Perme *Score* fue un instrumento desarrollado inicialmente para evaluar la movilidad funcional en la UCI, ha demostrado un gran potencial para su uso en diferentes escenarios. Es un instrumento que permite medir la movilidad funcional de forma rápida, objetiva y específica, además de considerar condiciones extrínsecas al paciente.

## Footnotes

1 Cotización del dólar estadounidense = R$ 5,84, el 01/02/2025.
